# PAU-SA: A Synthetic Aperture Interferometric Radiometer Test Bed for Potential Improvements in Future Missions

**DOI:** 10.3390/s120607738

**Published:** 2012-06-07

**Authors:** Isaac Ramos-Perez, Adriano Camps, Xavi Bosch-Lluis, Nereida Rodriguez-Alvarez, Enric Valencia-Domènech, Hyuk Park, Giuseppe Forte, Merce Vall-llosera

**Affiliations:** Remote Sensing Lab., Department Signal Theory and Communications, Universitat Politecnica de Catalunya (UPC) Campus Nord, Bldg D3, E-08034 Barcelona, Spain; E-Mails: camps@tsc.upc.edu (A.C.); xavier.bosch@tsc.upc.edu X.B.-L.;; nereida@tsc.upc.edu (N.R.-A.); valencia@tsc.upc.edu (E.V.-D.); park.hyuk@tsc.upc.edu (H.P.); giuseppe.forte@tsc.upc.edu (G.F.); merce@tsc.upc.edu (M.V.)

**Keywords:** microware, interferometric radiometer, calibration, soil moisture and ocean salinity, SMOS, Passive Advanced Unit Synthetic Aperture (PAU-SA)

## Abstract

The Soil Moisture and Ocean Salinity (SMOS) mission is an Earth Explorer Opportunity mission from the European Space Agency (ESA). Its goal is to produce global maps of soil moisture and ocean salinity using the Microwave Imaging Radiometer by Aperture Synthesis (MIRAS). The purpose of the Passive Advanced Unit Synthetic Aperture (PAU-SA) instrument is to study and test some potential improvements that could eventually be implemented in future missions using interferometric radiometers such as the Geoestacionary Atmosferic Sounder (GAS), the Precipitation and All-weather Temperature and Humidity (PATH) and the Geostationary Interferometric Microwave Sounder (GIMS). Both MIRAS and PAU-SA are Y-shaped arrays with uniformly distributed antennas, but the receiver topology and the processing unit are quite different. The purpose of this work is to identify the elements in the MIRAS's design susceptible of improvement and apply them in the PAU-SA instrument demonstrator, to test them in view of these future interferometric radiometer missions.

## 1. Introduction

Soil Moisture (SM) and Sea Surface Salinity (SSS) may seem to be unconnected, but both variables are intrinsically linked to the Earth's water cycle and the climate system. Thanks to the technological advances, the interest of the scientific community in remotely measuring SSS and SM has increased in the last years, spending much effort in this direction. The three current and planned Earth observation missions focused in these variables are: the MIRAS instrument aboard the SMOS mission of the European Space Agency (ESA) launched on November 2, 2009 [1], the Aquarius radiometer from National Aeronautics and Space Administration (NASA) aboard the Satelite de Aplicaciones Cientificas (SAC-D) spacecraft of the Comisión Nacional de Actividades Espaciales (CONAE) [2] launched on June 10, 2011, and the Soil Moisture Active and Passive (SMAP) mission of NASA [3] to be launched in 2014. All three missions carry an L-band microwave radiometer as primary instrument. A microwave radiometer is an instrument that measures the spontaneous electromagnetic radiation emitted by all bodies at a physical temperature different from 0 Kelvin. Microwave radiometers were first used in radio-astronomy in the 1930s [4]. In [5] the Passive Advanced Unit for ocean monitoring (PAU) project was proposed aiming at demonstrate that collocated measurements of sea brightness temperature and reflected Global Navigation Satellite System Reflectrometry (GNSS-R) signals can result in a significant improvement of the retrieved SSS. Its scientific goals are to perform ocean monitoring by passive remote sensing to improve the knowledge of the relationship of the GNSS-R signal with the sea state, and to improve the knowledge of the relationship between the L-band brightness temperature and the sea state. To accomplish these goals, the PAU sensor consists of three instruments that operate in a synergetic way:

- an L-band radiometer to measure the brightness temperature,
- a reflectometer to measure the sea state using reflected Global Positioning System (GPS) opportunity signals, sharing the Radio Frequency (RF) front-end, with the radiometer, and
- an Infrared Radiometer (IR) to measure the physical surface temperature.

The PAU project is also a test-bed of new technological demonstrators such as real aperture radiometers with digital beamforming and polarization synthesis [7], fully-digital synthetic aperture radiometers, *etc*. To perform this, a ground-based instrument demonstrator PAU-SA has been designed, implemented, and tested to validate these possible improvements. This work is organized as follows: in order to understand better these improvements, some basic concepts on interferometric radiometry are reviewed in Section 2. The interested reader is referred to [8,9] for a detailed review. Section 3 provides an overview of the PAU-SA instrument, and describe in detail the instrument itself. Section 4 describes briefly the processing steps of the PAU-SA data. The interested reader is referred to [10] for more details. Section 5 presents an comparison between the PAU-SA and the MIRAS instruments to illustrate which improvements and new techniques have been implemented and tested, and their rationale. Finally, Section 6 presents some measurements performed, and Section 7 summarizes the main conclusions and the lessons learned.

## 2. Basic Concepts on Interferometric Radiometry

**Figure 1.**
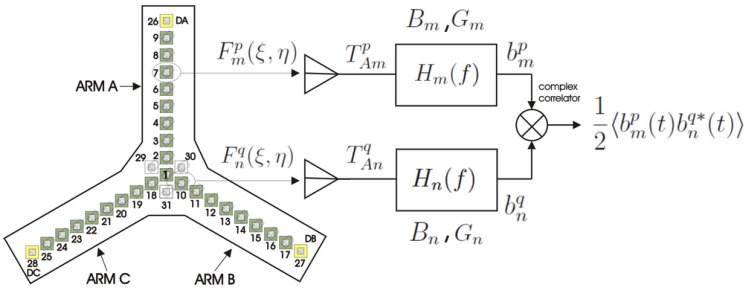
Sketch of the fundamental operation of an interferometer: each pair of receiving channels and a complex correlator form a baseline that measures a sample of the visibility function from [6].

The fundamental operation of a synthetic aperture radiometer is the complex cross-correlation of the signals collected by each pair of channels (antenna + receiver), or baseline as shown in Figure 1.

The variables in Figure 1 are: $F_{m,n}^{p,q}(\xi ,\eta )$ the normalized antenna patterns of channels *m* and *n*, at *p* and *q* polarizations, (*ξ, η*) = (sin *θ* cos *φ*, sin *θ* sin *φ*) are the direction cosines defined with respect to the X and Y axes, *T_Am_*,_n_ is the antenna temperature, *H_m,n_*(*f*) is the frequency response, *G_m,n_* is the power gain of the channel, *B_m,n_* is the equivalent noise bandwidth, $b_{m,n}^{p,q}$ analytical signal collected by each channel, and 〈 〉 is the expectation operator implemented in practice as a time average. A synthetic aperture radiometer measures the cross-correlations between all pair of signals, collected by the antennas. The auto-correlation and the cross-correlation of the signals at each receiver output are defined as:

(1) $$\frac{1}{2}\langle {\left|b_{m,n}^{p}(t)\right|}^{2}\rangle ≜k_{B}\cdot B_{m,n}\cdot G_{m,n}\cdot (T_{Am,n}^{p,q}+T_{\text{REC}m,n})$$

and

(2) $$\frac{1}{2}\langle b_{m}^{p}(t)b_{n}^{q*}(t)\rangle ≜k_{B}\cdot \sqrt{B_{m}\cdot B_{n}}\cdot \sqrt{G_{m}\cdot G_{n}}\cdot V_{mn}^{pq}(u_{mn},v_{mn})$$

where *k_B_* is the Boltzmann constant, *T_RECm,n_* is the equivalent receiver's noise temperature, and $V_{mn}^{pq}$ is the visibility function defined in the spatial frequency (baseline) (*u_mn_, v_mn_*) ≜ (*x_n_ − x_m_, y_n_ − y_m_*)/λ_0_ that depends on the difference of the antenna positions normalized to the wavelength, being λ_0_ = *c*/*f*_0_. According to Equation (2), the visibility function with units of Kelvin can be derived as a sample of the cross-correlation function:

(3) $$V_{mn}^{pq}(u_{mn},v_{mn})=\frac{1}{k_{B}\sqrt{B_{m}\cdot B_{n}}\sqrt{G_{m}\cdot G_{n}}}\frac{1}{2}\langle b_{m}(t)b_{n}^{*}(t)\rangle$$

Assuming that the collected signals are stationary random processes (thermal noise radiation) that fulfill the ergodicity property, narrow-band, spatially uncorrelated, and that the distance between antennas is much larger than the wavelength, Equation (3) can be computed in practice using the cross-correlation (time average) of the in-phase and quadrature components $(b_{m,n}^{p,q}=I_{m,n}^{p,q}(t)+jQ_{m,n}^{p,q}(t)):V_{mn}^{pq}(u_{mn},v_{mn})=<I_{m}^{q}I_{n}^{p}>+j<Q_{m}^{q}I_{n}^{p}>$.

From the Van-Cittert Zernicke theorem [11], the visibility function can be related to the brightness temperature distribution [12] as:

(4) $$V_{mn}^{qp}(u_{mn},v_{mn})=\frac{1}{\sqrt{\Omega_{m}\Omega_{n}}}\int \int_{\xi^{2}+\eta^{2}\leq 1}\frac{T_{B}^{qp}(\xi ,\eta )-T_{\text{rec}}\delta_{qp}}{\sqrt{1-\xi^{2}-\eta^{2}}}F_{m}^{q}(\xi ,\eta )F_{n}^{p*}(\xi ,\eta )\cdot {\tilde{r}}_{mn}\left(-\frac{u_{mn}\xi +v_{mn}\eta }{f_{0}}\right)e^{-j2\pi (u_{mn}\xi +v_{mn}\eta )}d\xi d\eta$$

where

- 𝛀*_m,n_* are the equivalent solid angles of the antennas,
- $T_{B}^{qp}(\xi ,\eta )$ is the *T_B_ of* the scene at*p* − *q* polarization [13] (*E_p_* and *E_q_* being the electric fields at *p* and *q* polarizations),
- *T_rec_* is the physical temperature of the receiver (the so-called Corbella's term) [12],
- *δ_pq_* is the Kronecker's delta function: *δ_pq_* = 1if *p* = *q* and *δ_pq_* = 0 if *p* ≠ *q*,
- ${\tilde{r}}_{mn}\left(-\frac{u_{mn}\xi +v_{mn}\eta }{f_{0}}\right)$ is the so-called fringe-washing function. This term is related to the limited bandwidth and the frequency response of the filters in the two receivers forming the baseline, being ${\tilde{r}}_{mn}≜\frac{e^{-j2\pi f_{0}t}}{\sqrt{B_{m}B_{n}}}\int_{0}^{\infty }H_{m}(f)H_{n}(f)*e^{j2\pi f_{t}}d_{f}$, and
- $1/\sqrt{1-\xi^{2}-\eta^{2}}$ is the obliquity factor.

In addition, Equation (4) is normalized by the term $k_{B}\cdot \sqrt{B_{m}B_{n}}\cdot \sqrt{G_{m}G_{n}}\cdot \sqrt{\Omega_{m}\Omega_{n}}$ to ensure that the visibility function has units of Kelvin.

### 2.1. Ideal Situations

A simplified version of Equation (4) is analyzed. Given the narrow bandwidth of PAU-SA (2.2 MHz) and the reduced dimensions of the array (being Δ*r* ≈ 2.4 m the maximum distance between two antennas of the array), the fringe-washing function becomes:

(5) $${\tilde{r}}_{mn}\left(-\frac{u_{mn}\xi +v_{mn}\eta }{f_{0}}\right)\approx 1$$

Assuming that all the antenna patterns are identical *F_m_* = *F_n_*, and the constant terms are normalized to the unity, the relationship between the visibility function *V_mn_*, (Equation (7)) and the so-called modified brightness temperature (Equation (6)) is given by Equation (8):

(6) $$T(\xi ,\eta )=\frac{T_{B}(\xi ,\eta )}{\sqrt{1-\xi^{2}-\eta^{2}}}{\left|F_{n}(\xi ,\eta )\right|}^{2}$$

(7) $$V_{mn}=\int \int_{\xi^{2}+\eta^{2}\leq 1}\left[\frac{T_{B}^{qp}(\xi ,\eta )}{\sqrt{1-\xi^{2}-\eta^{2}}}{\left|F_{n}(\xi ,\eta )\right|}^{2}\right]e^{-j2\pi (u_{mn}\xi +v_{mn}\eta )}d\xi d\eta$$

In these conditions, the modified brightness temperature can be recovered by means of the inverse Fourier transform of the visibility samples.

(8) $$T(\xi ,\eta )={ℱ}^{-1}[V_{mn}]$$

## 3. PAU-SA Description

### 3.1. PAU-SA Instrument Overview

**Figure 2.**
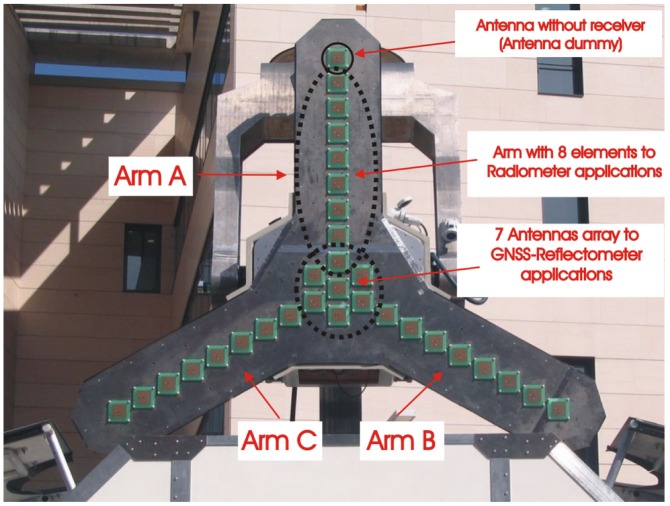
Picture of PAU-SA's array without radome from [6].

Figure 2 shows the PAU-SA instrument without the radome. It is composed by a Y-shaped array of 25 antennas for radiometric applications: 8 antennas uniformly spaced every *d* = 0.816*λ* per arm plus the one in the center. An additional antenna connected to a matched load, but without receiver or antenna dummy is included at the end of each arm to improve the antenna pattern similarity Moreover, in the original design, the reflectrometer part (PAU-SA GNSS-R) was going to use the 4 central antennas plus 3 additional ones (7 antennas in total) to create a steerable array to point to the Global Navigation Satellite System (GNSS) signal specular reflection points. Nowadays, this part has been simplified by a single additional antenna plus a GPS receiver front-end and data logger for off-line processing.

**Figure 3.**
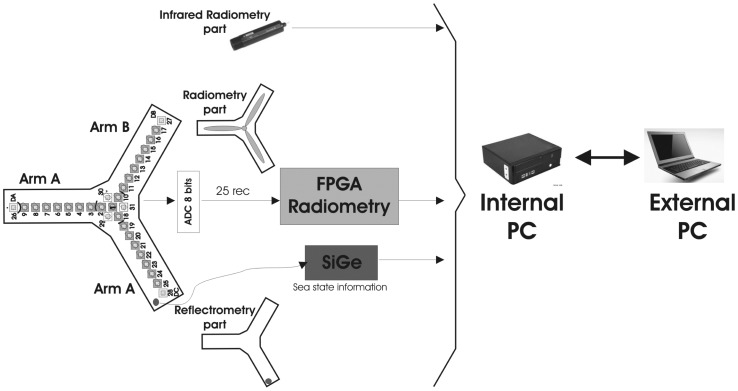
Global view of the PAU-SA's architecture from [6].

Each dual-polarization RF front-end is integrated behind a dual polarization patch antenna. The three main features of this receiver are: simultaneous dual polarization (V & H), frequency of operation (GPS L1 band) the same for both instruments (radiometer, and reflectometer), a three-stage down-converter, and a Local Oscillator (LO) inside each down-converter generated from a 10 MHz reference master clock common to all receivers. In this way, since the LO is generated inside each down-converter, their noises are uncorrelated from receiver to receiver and do not introduce a correlation offset. Each receiver translates the input signal from 1,575.42 MHz to an intermediate frequency of 4.309 MHz with a bandwidth of 2.2 MHz and an approximated gain of 110 dB. The differential Intermediate Frequency (IF) signal is sent to the Analog to Digital Converter (ADC) array unit through twisted pair (RJ45 grade 5) cables. These signals are digitalized at eight bits using IF sub-sampling techniques with a sample frequency of *F_s_* = 5.757 MHz. These data enters into a Virtex 4 Field Programmable Gate Array (FPGA) for digital In-phase and Quadrature (I/Q) down-conversion and digital filtering using 8 bits. Then, all possible cross-correlations between antenna pairs at each polarization are simultaneously performed at 1 bit only, using the sign bit, with maximum integration times multiple of 1 s. At the same time the 8 bit digitalized signals are squared and accumulated to compute the signal power, instead of using power detector diodes, as in MIRAS. Using digital techniques it is possible to eliminate 3 possible error sources: quadrature errors in the I/Q analog demodulators, thermal drifts in the Power Measurement System (PMS) due to the diodes' thermal stability used in analog systems, and quasi-perfect matching of the frequency responses for all receiver's using a digital filter (the only differences are due to the RF analog filters, which are wider). Once the correlation matrix is calculated, the data is sent via a Universal Asynchronous Receiver/Transmitter (UART) interface from the FPGA to the internal Personal Computer (PC) for an off-line post-processing in the external PC Figure 3. Due to the high volume of data, it is not possible to process it inside the FPGA. The external PC performs three tasks: (1) to undertake the calibration process needed before each acquisition process; (2) to do the off-line post-processing for the image reconstruction; and (3) to record the data for off-line post-processing.

The detailed description of the calibration approach is described in [10]. For calibration purposes, a noise distribution network distributes correlated noise into all receivers at two different noise levels (hot and warm) for phase calibration. Moreover, an uncorrelated load is used inside each receiver for correlation offset estimation.

### 3.2. PAU-SA Instrument Description

**Figure 4.**
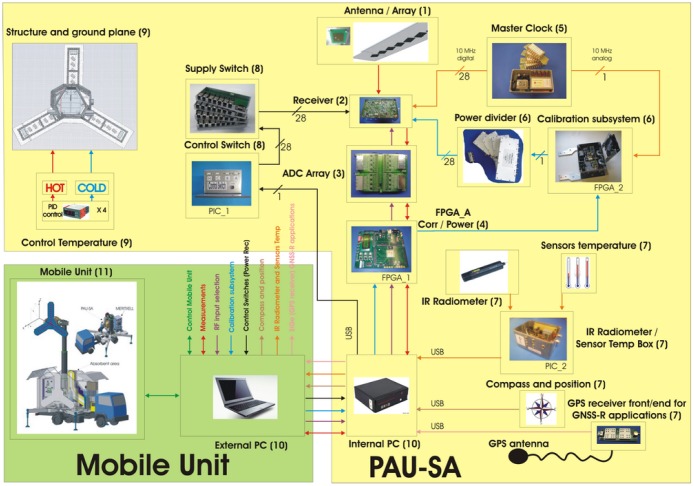
PAU-SA's system block diagram indicating the interface connections between different modules from [6].

An overview of the PAU-SA instrument is first given in Section 3 to provide a global understanding of the system. Figure 4 shows the main block diagram where the main modules are interconnected, which will be described in detail in the next subsections. These modules have been separated in two main blocks: the PAU-SA instrument and the mobile unit, adopting a centralized control through the external computer. This basically makes two control tasks: to control the motion of the articulated arm, and to control the internal computer that at the same time controls the devices located inside the PAU-SA instrument.

#### 3.2.1. Antenna Array

The first element of the instrument are the antennas. These are square patch antennas [14] with dielectric air to resonate at 1,575.42 MHz (GPS L1 frequency). The patch has been printed on 0.6 mm FR4 substrate offering simplicity and low production costs. The main parameters are: matching better than −22 dB in each polarization (V/H), low ohmic losses *η*_𝛀_ = 0.98, and half-power beam width of 60°. The antenna array consists of 25 antennas distributed over a Y-shaped array, with 8 antennas per arm, and a central one for radiometry applications. Three extra dummy antennas (with no receiver connected) are placed at the end of each arm to improve the antenna voltage patterns similarity as analyzed in [15] and [16]. Moreover, three antennas are placed around the central one for future GNSS-R applications [17] (Figure 2). Nowadays, this part has been replaced by a ceramic patch GPS Left Hand Circular Polarization (LHCP) antenna and a GPS L1 receiver [18] connected to the internal PC for acquisition of the raw GPS data. A maximum distance of $\lambda /\sqrt{3}(d=0.577\lambda )$ between antennas is required to avoid aliasing in the image reconstruction. However, due to the physical dimensions of the receivers and antennas, the minimum feasible distance in PAU-SA is 15.5 cm (d = 0.816 *λ*), thus some aliasing will affect the borders of the recovered image.

#### 3.2.2. Receiver

**Figure 5.**
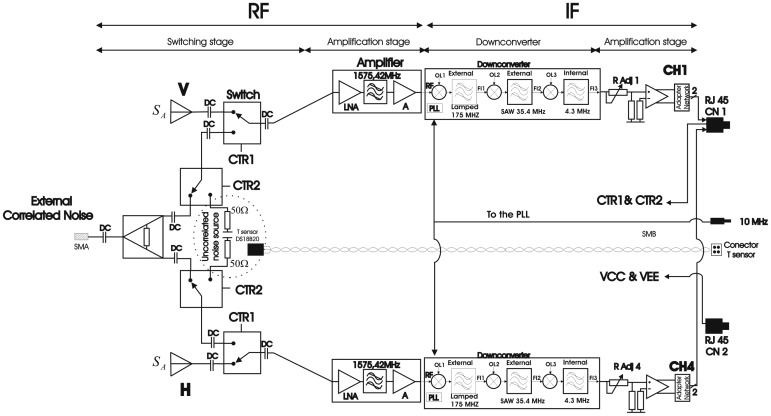
PAU-SA's receiver block diagram uses two TPR topologies, one per polarization (V & H) from [6].

The receiver has been designed to combine two different sub-systems: an L-band radiometer and a GNSS-Reflectometer. Since the reflectometer part requires continuous data acquisition, the input cannot be chopped. Therefore, Total Power Radiometer (TPR) topology for each vertical and horizontal polarization has been selected (Figure 5). Each chain is individually amplified, down-converted, and amplified again to allow an 8-bit quantification for later digital processing (cross-correlation, power detection, and calibration). Since the noise introduced by the amplifiers is uncorrelated (not considering the cross-talk), it vanishes when the output signals are cross-correlated.

To improve the Alias-Free Field Of View (AF-FOV), the receiver has been implemented in reduced dimensions (11 cm × 7 cm × 3 cm). Taking into account that the operating frequency is the L1 GPS band, many already existing commercial off-the-shelf (COTS) components have been used for the implementation of this receiver, such as the Zarlink GP2015 GPS front-end as down-converter [19], Low Noise Amplifier (LNA), and Surface Acoustic Wave (SAW) filters. The receiver was carefully designed to preserve symmetry, minimize cross-talk, and interconnection routes, while maintaining equal delays for all paths, being divided in two parts: RF and IF stages. Due to space limitations the two stages have been implemented in two boards one over the other, using different substrates according to the frequency of each stage.

##### 3.2.2.1. RF Stage

In order to ensure a constant impedance of the transmission lines, the RF stage has been implemented with ROGERS 4003 0.8 mm thickness substrate. Its structure consists of two parts, the switching stage and then an amplification stage. The main objective of the RF stage is to achieve a gain of at least 30 dB over the input signals, keeping the Noise Figure (NF) as low as possible. This is necessary to work in the linear region of the down-converter module located in the IF stage. The matching of each input/output ports have to be at least −22 dB. The required isolation between antenna signal and calibration (correlated and uncorrelated) signals has been experimentally determined to be better than −80 dB. Finally, the cross-talk between adjacent TPR have to be better than −40 dB.

The switching stage is controlled through CTR1 and CTR2 signals by the Control Unit (CU) implemented in the FPGA. It switches between three different states: the antenna measurements, calibration with external correlated signals (either noise or Pseudo-Random Noise (PRN) signal), or calibration with uncorrelated noise generated by an internal 50 𝛀 matched load. Usually this stage is connected to antenna acquisition, necessary for GNSS-R applications. The antenna input is connected directly to the patch antenna through a 50 𝛀 cable. Calibration with correlation signal is required to compensate different phases and amplitudes among channels. To implement this structure two switches model RSW-2-25P by MiniCircuits have been used, one for each radiometer, increasing the isolation between correlated noise and antenna signal. Once selected, the signal is amplified to adjust the antenna input power (∼−110 dBm) to the down-converter linear behavior power margin. The total RF gain is ∼33.3 dB, obtaining an input power level for down-converter of ∼−76.7 dBm, within the linear behaviour, and the total NF of 2.2 dB.

##### 3.2.2.2. IF Stage

The main goal of the IF stage is to shift the RF signal to IF using a superheterodyne receiver or down-converter. Moreover this stage pre-amplifies the IF signal and transmits it to the ADC module through an Ethernet cable. This stage has been implemented with FR4 substrate. The output of each RF chain is interconnected by means of a semi-flexible cable to the input of each down-converter located in this stage. It mainly consists of the translation from 1,575.42 MHz to 4.309 MHz with a bandwidth of 2.2 MHz amplifying the RF signal by approximately 52 dB. The down-converter requires a 10 MHz Transistor–Transistor Logic (TTL) clock signal. This signal is distributed by a coaxial cable and it is used to internally synthesize the different LOs by means of the Phase Lock Loop (PLL)s. Usually these devices provide the output at IF, digitized at 2 bits (sign and magnitude) enough to recover the Coarse Acquisition (C/A) code, used in GPS applications. Originally, it was intended to determine the signal's power using just 1 bit as in [20] using the Nemerix NM1100, but this coarse sampling was found totally unacceptable for radiometric applications. Therefore, the Zarlink GP2015 GPS down-converter was used instead, since it has an analog output test that can be sampled. A commercial GPS down-converter can be used for radiometric applications if it has a linear behavior. However, all commercial GPS receivers have an Automatic Gain Control (AGC) circuitry that modulates the gain depending on the input signal to achieve a constant power level at its output. The GP2015 AGC control has been tuned forcing a differential voltage between its pins. In this case, for input levels from −85 dBm to −60 dBm it behaves linearly, with a gain of 52 dB, and with a maximum linearity error of 0.25 dB (Note that the dynamic range of the radiometer is actually much smaller (less than 3 dB) for which the linearity 0.2%).

At the receiver's output the signals are centered around *f_IF_* = 4.309 MHz, with a *B* = 2.2 MHz bandwidth. Each down-converter output is then amplified using a NE592D video amplifier, with fixed gain and differential output in order to cancel the common mode errors in the transmission to the ADCs array using Ethernet category 5 cables. Due to component tolerances it is necessary to perform manually a relative adjustment of each channel by means of the voltage divider, between the down-converter, and the video amplifier (Figure 4). The goal of the voltage divider is to adjust the gain to obtain a 110 mV rms signal at the receiver's output, to match the ADC input range. The gain of each stage should have a maximum error of ±1 dB to allow the correction in the digital process part. Due to the space limitations, the correlation function is implemented digitally outside the receiver, using a Virtex 4 LX60 FPGA [21]. The analog signals are sent to an external ADCs array to be digitalized. To do this, the output of each video amplifier is connected to a matching network to match its output impedance to the 150 𝛀 of the twisted pair (RJ45 grade 5 cable). This connector is shared by four differential outputs at 4.309 MHz, two RF switch control inputs, and two power supplies. Due to the amount of pairs, two RJ45 connectors are needed. This method minimizes considerably the total number of cables connected to the receiver, and at the same time provides a high isolation against possible interferences.

##### 3.2.2.3. PAU-SA's Receiver Implementation

**Figure 6.**
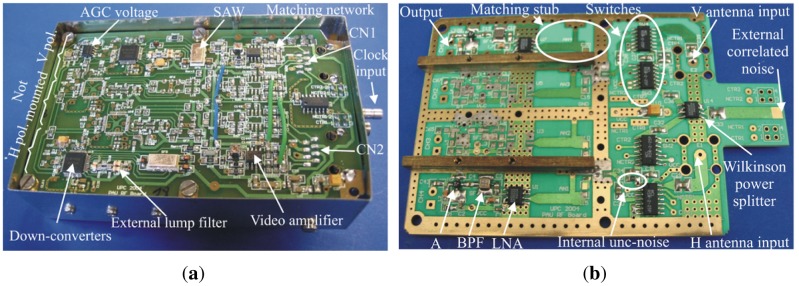

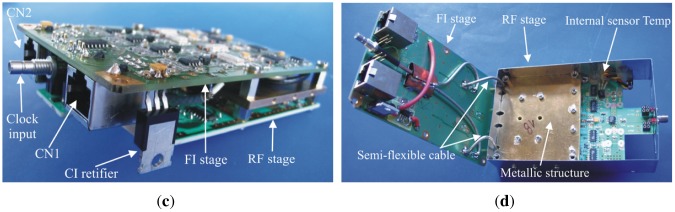
(**a**) PAU-SA's receiver IF stage view with box, and (**b**) RF stage view without box, (**c**) assembled stages to minimize the required area, and (**d**) interconnection stages (from [6]).

The design of PAU-SA's receiver has been based on PAU-Real Aperture (PAU-RA) [7,22], the real aperture version of the PAU instruments family. However, due to the large number of receivers needed in the PAU-SA instrument and the limited number of I/O pins in the FPGA to implement the correlations, it was not possible to use the same pseudo-correlation topology, and a total power radiometer topology was selected instead of a pseudo-correlation one. As it can be seen in Figure 6(a) and 6(b), PAU-SA's receiver has only half of the components mounted in the IF and RF stages.

Once the RF and IF boards are assembled separately, they have been assembled and interconnected, as shown in Figure 6(c) and 6(d).

#### 3.2.3. Digital Sub-Systems

The sub-systems implemented in the FPGA are:

- In-phase (I) and quadrature (Q) demodulation of the receivers' output digital signals coming from the array of 8 bits ADC),
- Digital Low Pass Filter (LPF) at 8 bits,
- Power estimation system of the 50 receivers' output signals (25 receivers × 2 polarizations @ 8 bits).
- Correlation unit of the three correlation matrices (V, H, VH) @ 1 bit, and
- Communication protocol and control with a PC, and data collection.

**Figure 7.**
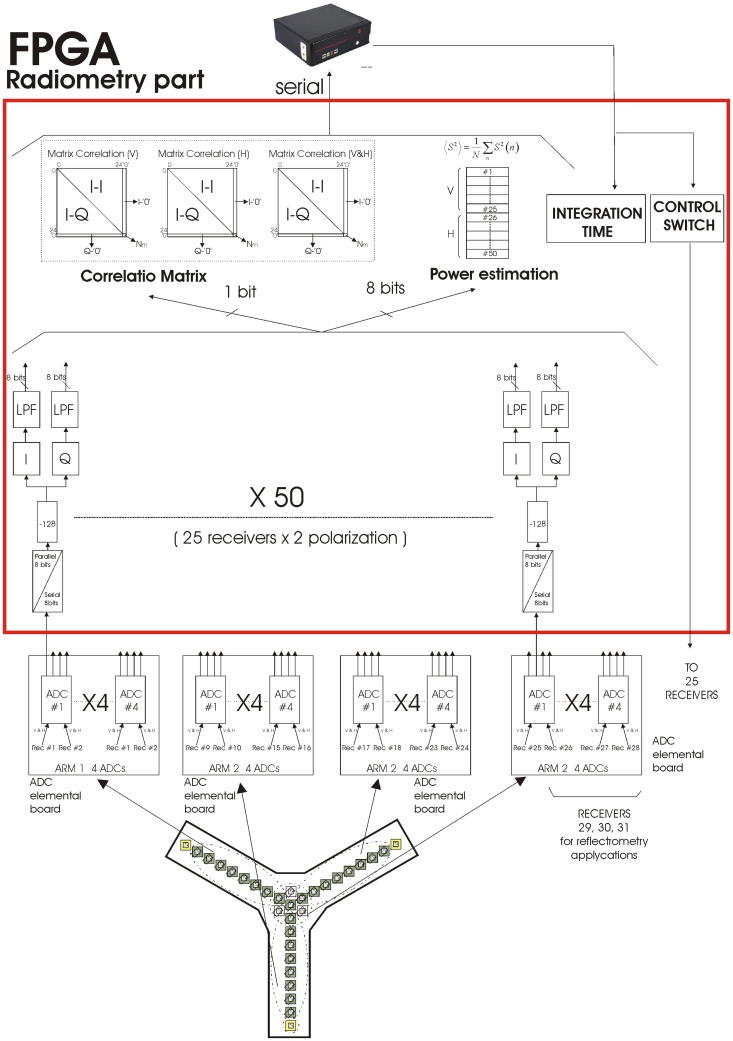
Overview of the sub-systems implemented in the FPGA (radiometer part) and peripherals in PAU-SA from [6].

The FPGA device used in the design is the Xilinx Virtex-4 LX 60 distributed. Figure 7 shows the block diagram of the main sub-systems previously discussed and their implementation in the FPGA. Other peripherals connected to the FPGA, such as an external PC, and an array of ADCs are also shown for clarity. Before start explaining the design of the sub-systems implemented in a FPGA, it is necessary to analyze the analog-to-digital converter. Since there are 25 receivers, and each one has a dual polarization antenna with IF differential output, a total of 50 analog differential channels are connected to the array of ADCs. Moreover the receivers need two unipolar common control signals (CTRL1 and CTRL2) to select the different RF inputs. These two control signals are sent by the external PC to the receivers through the FPGA first, and then via the ADC array. The IF output signals are down-converted at a central frequency of 4.309 MHz with a bandwidth of 2.2 MHz, and a dynamic range of the output voltage of 1 *V_rms_*.

##### 3.2.3.1. Sampling Frequency

**Figure 8.**
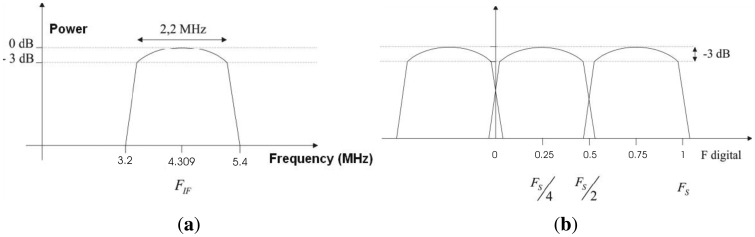
(**a**) Frequency domain of input signal, and (**b**) frequency domain of input signal digitalized. Pictures from [6].

The fundamental requirement of a sequential electronic block is the frequency of operation. In the case of the ADC, the frequency of operation determines the sampling frequency. In the PAU radiometer the receiver has a 4.309 MHz intermediate frequency, and then it is digitized to translate the frequency to baseband with the Digital Down Converter (DDC). The Nyquist sampling theorem states that the sampling frequency should be at least twice the highest frequency components in the signal. This means that to sample a 4.309 MHz signal, a minimum 8.618 MHz sampling frequency is required. A higher sampling frequency implies a higher data rate, and the DDC function will be more complex to implement in the FPGA because the input signal of DDC should be multiplied by a cosine function using all the 8 bits. Another option is the so called “band-pass sampling”. With this technique a smaller sampling frequency can be used, thus reducing the data rate, while simplifying the implementation of the DDC. The “band-pass sampling” technique is described in the following paragraphs. If the input signal of DDC is multiplied by cos(𝛀*n*), where 𝛀 = 2*πF_digital_* is the carrier frequency, and *F_digital_* is the digital frequency with a value of 0.25 (Figure 8(a)), the input signal results:

(9) $$\begin{array}{cc} \cos(\frac{\pi }{2}\cdot n)=1,0,-1,0,\ldots & \text{with}\;n=0,1,2,3,\ldots \end{array}$$

This means that now, the input signal has to be multiplied by 1,0,-1,0 thus simplifying the numerical computation. Now, it is possible to compute the sample frequency of the ADC, considering the sampling frequency of ADC (*F_S_*) and the frequency of output signal from receiver (*F_IF_*), the resultant frequency is:

(10) $$F_{\text{FINAL}}=F_{S}-F_{IF}$$

Taking *F_FINAL_* = *F_S_*/4 the *F_S_* results:

(11) $$F_{S}=4\cdot F_{\text{FINAL}}=4\cdot (F_{S}-F_{IF})\Rightarrow F_{S}=\frac{4}{3}F_{IF}$$

(12) $$F_{IF}=4.309\text{MHz}\Rightarrow F_{S}=5.745\text{MHz}$$

Thus, the output signal of the ADC is centered at the frequency *F_FINAL_* = 1.436 MHz, as shown in Figure 8(b).

##### 3.2.3.2. I/Q Demodulation Unit

**Figure 9.**
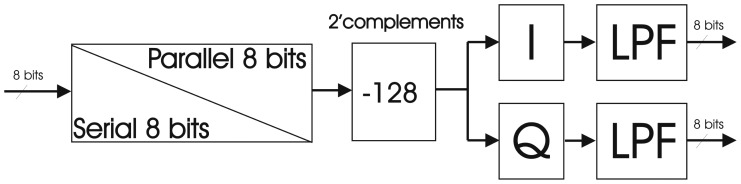
Block I/Q demodulation unit from [6].

Before calculating the correlation matrices, it is necessary to down-convert the digital signals of the receivers to baseband and obtain their in-phase (I) and quadrature (Q) components. This section briefly presents the theoretical formulation and then explains the main blocks. The I/Q demodulation unit is composed of four blocks as shown in Figure 9: serial-to-parallel converter, two's complement, I/Q demodulation, and a selective LPF. The serial to parallel block is necessary to convert serial output of the ADCs forced by the limited number of pins in the FPGA to parallel for easier internal operations. Therefore the ADC device has been chosen with serial output and a sampling frequency of 5.745 MHz. Since the raw data that comes from the ADC is not suitable for data processing due its offset (128), this offset has to be subtracted. This offset is related to the fact that the ADC output gives a binary quantification of the analog signal between 0 and 255 (8 bits). Hence, it is necessary to subtract this offset to have symmetrical positive and negative values at the same time, convert the data into 2's complement to simplify the signal processing. The two main blocks are I/Q demodulation and the LPF, explained in detail in this section.

Once the receiver's signal has been digitized the output has the following expression:

(13) $$S(n)=\mathbb{R}\{(i(n)+jq(n))\cdot e^{j\Omega n}\}$$

where 𝛀 = 2*πF_digital_* is the carrier frequency. The above given expression can be rewritten as:

(14) S(n)=i(n)cos(Ωn)−q(n)sin(Ωn)

Taking into a count the previous equation, it is possible to obtain the *I*(*n*) and *Q*(*n*) components multiplying *S*(*n*) by a cosine and a sine respectively, and low-pass filtering them:

(15) $$I(n)=S(n)\cos(\Omega n)=\frac{1}{2}i(n)+\frac{1}{2}(i(n)\cos(2\Omega n)-q(n)\sin(2\Omega n))$$

(16) $$Q(n)=S(n)(-\sin(\Omega n))=\frac{1}{2}q(n)-\frac{1}{2}(i(n)\sin(2\Omega n)+q(n)\cos(2\Omega n))$$

If these expressions are particularized to the digital frequency (*F_digital_* = 0.25), the product of *S*(*n*) by a cosine and a sine is equivalent to multiply *S*(*n*) by a periodical sequence with period four:

(17) $$\begin{array}{ccc} \cos(2\pi F_{\text{digital}}n)=\cos(\frac{\pi }{2}n)=1,0,-1,0,\ldots & \text{with} & n=0,1,2,3,\ldots \end{array}$$

(18) $$\begin{array}{ccc} -\sin(2\pi F_{\text{digital}}n)=-\sin(\frac{\pi }{2}n)=0,-1,0,1,\ldots & \text{with} & n=0,1,2,3,\ldots \end{array}$$

**Figure 10.**
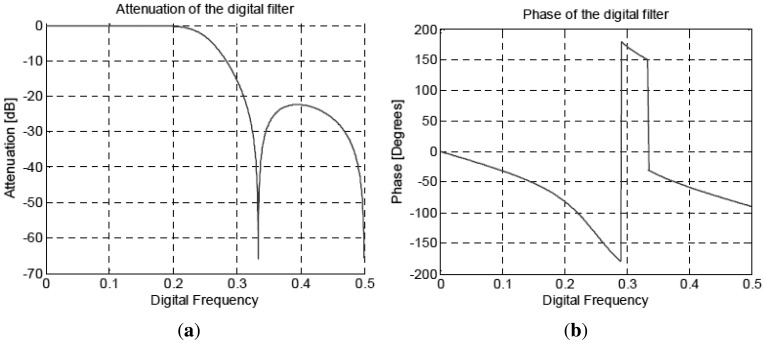
(**a**) Gain response of the IIR filter (**b**) Phase response of the IIR filter. Pictures from [6].

Working with the ADCs and using band-pass sampling, it is then possible to work with a specific digital frequency that minimizes the required hardware resources. In this particular case it is not necessary to implement a multiplier stage, but only to take a sample out of two with the corresponding sign. These modules are implemented with a multiplexer block and an inverter function. In Equations (15) and (16) it is possible to observe a low frequency in-phase and quadrature terms plus a high frequency contribution that must be eliminated by a LPF. The filter implemented is an Infinite Impulse Response (IIR) LPF with a cut-off frequency of 0.25 in the digital domain with an attenuation larger than 20 dB at the frequency of interest, and 6 dB of gain that compensates the 0.5 factor in the phase and quadrature Equations (15) and (16), (Figure 10(a)). The disadvantage is that the phase does not have a perfectly linear behavior (Figure 10(b)), and the group delay changes with frequency. However, this is not relevant, since all filters have exactly the same behavior, and uniformity is guaranteed across them, unlike with analog filters. These filters have been implemented only with elementary functions: delay blocks, adders and shift registers using the minimum FPGA resources.

#### 3.2.4. Power Measurement

To recover the normalized visibility function as derived from the correlation matrix (Equation (2)), an estimation of the system temperature is necessary for both polarizations. This means 25 (receivers) × 2 (polarizations) = 100 different power measures. To obtain this, each channel is considered as a total power radiometer and the signal's power is estimated as (Equation (19))

(19) $$<{\hat{S}}^{2}>=\frac{1}{N}\sum_{n}S^{2}(n)$$

Since our signal is thermal noise, the power measurement can be obtained either from the IF signal or from each of the I/Q components. For the sake of simplicity, the I and Q components have been used. To implement a power measurement system a multiplier and an adder are required. For this application the internally implemented FPGA functions are used.

#### 3.2.5. Digital Correlation Unit (DCU)

**Figure 11.**
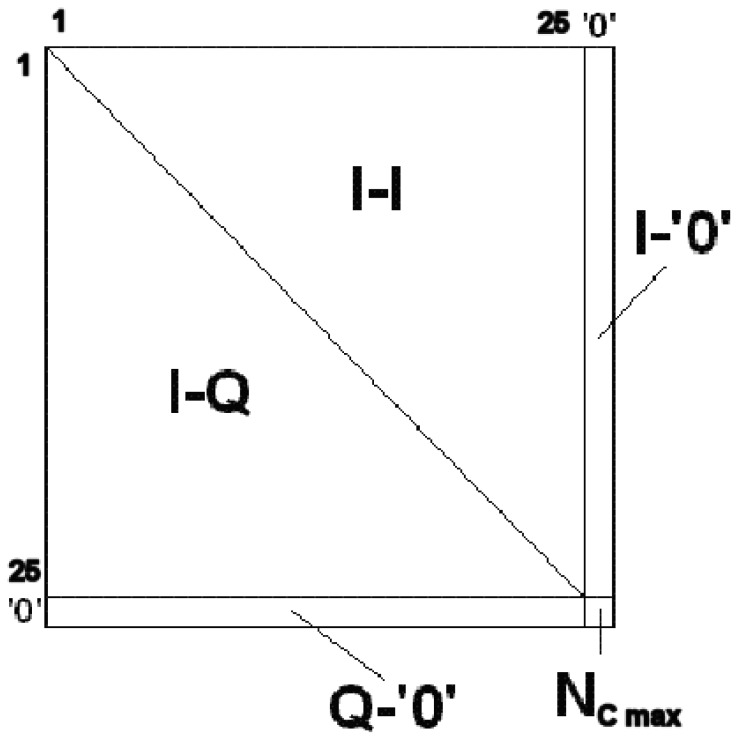
PAU-SA's correlation counts matrix *N_cm,n_* from [6].

**Figure 12.**
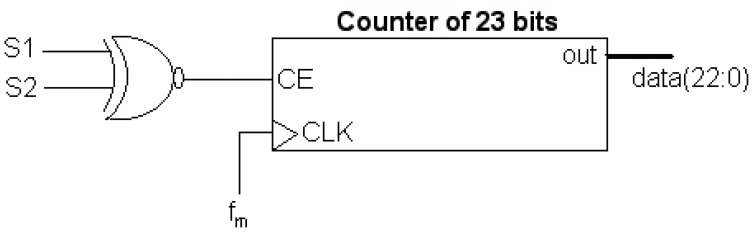
Elemental correlator block (from [6]).

The computation of the digital correlations and the power measurements are the two pre-processing steps implemented in the FPGA. The Digital Correlation Unit DCU measures the similarity between both signals using 1 bit (sign bit). It basically consists of counting the number of samples (*N_c_*) with the same sign between all possible baseline combinations. Moreover, it is also necessary to obtain the maximum number of samples (*N_cmax_*), which is proportional to integration time, so as to normalize and obtain the correlation matrix (c) in the post-processing. Since the sampling frequency (*F_S_*) is 5.745 MHz and that the maximum integration time (*T_int_*) is 1 s, then the maximum number of counts (*N_cmax_*) is given by Equation (26), requiring a 23 bits a 2^23^ ≃ 8.388 MS counter enough to count up to 5.745 MSamples. In this case, with 25 receivers and the *I* and *Q* demodulated components, the correlation matrix can be obtained (Figure 11). Recall that the correlations between the in-phase components (I-I) are the real part of the normalized visibility (*μ_r_*), and the correlations between in-phase and quadrature components (I-Q) correspond with the imaginary part of the normalized visibility (*μ_i_*). In the diagonal, the cross-correlation between the in-phase and quadrature components (I-Q) of the same signal are computed, but as expected due to the digital I/Q demodulation, they are zero. Basically, to implement the correlator block, two elementary components are required: a logic equality detector or XNOR gate, and a 23 bits counter, as shown in Figure 12.

To implement only one polarization matrix, 676 results of 23 bits are necessary. Due to limitations in the FPGA resources the internal Random Access Memory (RAM) memory has been used to implement the counters (676 × 3 = 2,026 counters). Also, since the frequency of operation is higher than the sampling frequency (*F_CLK_* = 103.41 MHz ≫ *F_S_* = 5.5745 MHz), it is possible to divide the sampling time in different slots, and reuse the hardware by a factor:

(20) $$r=\frac{T_{m}}{T_{\text{clok}}}=18$$

This value means that it is possible to do 18 internal operations until the next sample arrive. In our case the FPGA used has 160 independent RAM blocks to implement 3 correlation matrices, this means 53 blocks for each block. In this case, the required hardware reuse factor is:

(21) $$r=\frac{676\;\text{counters each matrix}}{53\;\text{RAM blocks each matrix}}=12.7$$

which is smaller than the maximum one (Equation (20)).

#### 3.2.6. Correlated Noise Unit

**Figure 13.**
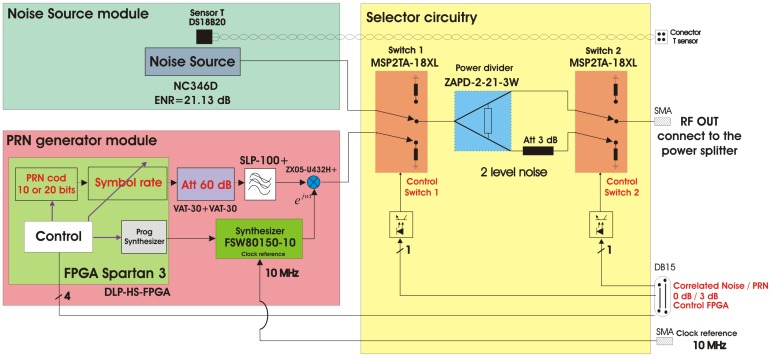
Correlated noise unit block diagram from [6].

Figure 13 shows the correlated noise diagram. It consist of three main blocks: two blocks for the signal generation (on the left), composed of thermal and pseudo-random noises, and a selection circuitry (on the right). Regarding the noise signals there are two different: a classical noise source, and a new technique based on using Pseudo-Random Noise PRN sequences, which can be selected using the selector block.

##### 3.2.6.1. Thermal Noise

The Classical noise source has been implemented with a NoiseCom noise source model NC346D with an Excess Noise Ratio (ENR) of 21.31 dB [23]. The ENR can be related with an equivalent temperature *T_n_* at the output of the noise source as:

(22) $$\begin{array}{cc} ENR_{dB}=10\log\left(\frac{T_{n}}{T_{0}}\right)-1 & \left[\text{dB}\right] \end{array}$$

where *T*_0_ is the reference temperature (290 K). In this case, the resulting *T_n_*:

(23) $$\begin{array}{cc} T_{n}=T_{0}\cdot \left(10^{\frac{ENR_{dB}}{10}}+1\right)=39.5\cdot 10^{3} & \left[\text{K}\right] \end{array}$$

##### 3.2.6.2. Pseudo-Random Noise (PRN)

Pseudo-Random Noise (PRN) sequences are signals with very long repetition periods that are used in a variety of applications, such as Code Division Multiple Access (CDMA) communications or positioning systems. They have a relatively flat spectrum over a bandwidth determined by the length of the sequence and the speed of the code or Symbol Rate (SR). Their spectra look like the noise spectrum, and the calibration of a microwave correlation radiometer (either interferometric or polarimetric) can benefit from these properties The SR parameter is used to determine the speed of the PRN code in order to control the bandwidth of the spectrum. The symbol rate is defined as the ratio of the bandwidth of the PRN signal (*B_PRN_*) and the receiver's low-pass equivalent bandwidth (*B*, Equation (24)). *B_PRN_* is related to the sequence duration (*τ_PRN_*) and the number of chips (a chip is like a bit, but it does not carry any information) *N_chips_* as shown in Equation (25).

(24) $$SR=\frac{B_{\text{PRN}}}{B}$$

(25) $$B_{\text{PRN}}=\frac{N_{\text{chips}}}{\tau_{\text{PRN}}}$$

The equivalent noise temperature of the PRN signal (*T_PRN_*) at the PRN generator module output is defined in terms of the PRN signal's amplitude (A): *P_PRN_* = *A*^2^/2 ≜ *k_B_* · *T_PRN_* · *B_PRN_*, where *P_PRN_* is the PRN signal power, and *k_B_* is the Boltzmann constant.

**Figure 14.**
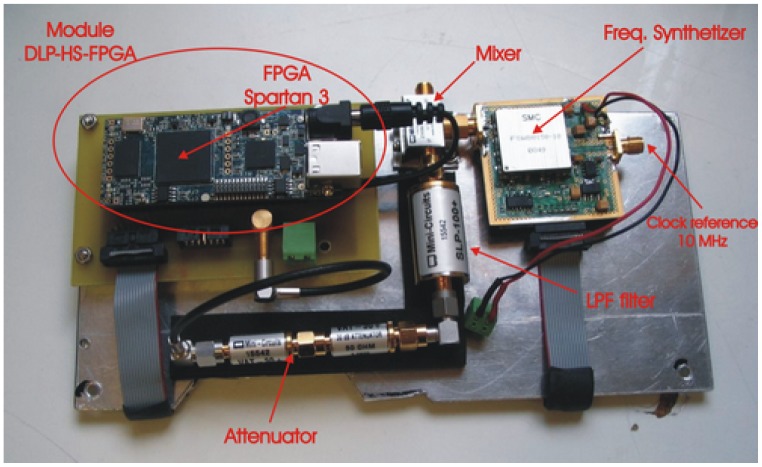
PRN generator module circuitry from [6].

The PRN is generated with a Linear Feedback Shift Register (LFSR) [24] as used for example in GPS applications. This part has been implemented in the FPGA model Spartan 3 of Xilinx using the module HLP-HS-FPGA [25] (Figure 14).

**Figure 15.**
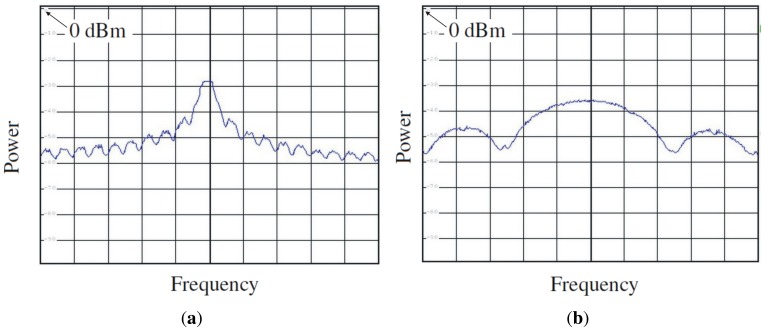
Spectrum analyzer acquisitions of different PRNs generated with different SRs. Central frequency of 1,575.42 MHz, 2 MHz/div, span = 20 MHz and 10 dB/div. (**a**) SR = 1, and (**b**) SR = 5 (from [6]).

The system has been designed to have the possibility to select between 10 or 20 order primitive polynomials which maximum-length to generate two pseudo-random sequences of length 2*^nbits^ −* 1, respectively. The LFSR can produce an output at different speeds by selecting the SR parameter. Internally the reference clock of 1.023 MHz is multiplied by the SR parameter to change the speed of the sequence. Taking as a reference the polynomial of order 10 with a length sequence of 1,023 chips, with SR = 1, the complete sequence is generated in 1,023 chips/1.023 MHz = 1 ms, with SR = 2, there are two complete sequences generated in the same time, and so on. With the 20th order polynomial with a length sequence of 1,048,575 chips, the sequence looks like more random, that is to say, with SR = 1, 1,048,575 chips/1.023 MHz = 1,025 ms is needed to repeat the sequence, and with SR = 2 half the time is needed, and so on. The output of the LFSR is baseband and it is internally modulated up to 80 MHz to be up-converted with the external mixer up to 1,575.42 MHz. Since the output of the digital signal is around 3.3 V, it has been necessary to put some attenuators in order not to saturate the receivers. In this case a 60 dB attenuator has been achieved cascading two attenuators of 30 dB model VAT-30 from MiniCircuits [26]. Then, this signal is low-pass filtered at 100 MHz with the filter model SLP-100+ from MiniCircuits [27] to eliminate possible spurious signals out of the band of interest. Finally the baseband signal is up-converted to the frequency of interest (L1) using the ZX05-U432H+ mixer from MiniCircuits [28]. To do this, the mixer needs a local oscillator at 1,575.42 MHz − 80 MHz = 1,495.42 MHz, which has been implemented using a surface mount frequency synthesizer model FSW80150-10 from Synergy Microwave Corporation [29]. This device is programmed at the beginning through the Spartan-3 FPGA. An analog reference clock of 10 MHz and 1 Vpp generated in the module Master clock is necessary in the synthesizer to feed the internal PLL. Figure 15 shows an acquisition with the spectrum analyzer where it is possible appreciate the PRN bandwidth in function of the SR parameter. The higher the SR, the higher the PRN bandwidth.

###### 3.2.6.2.1. Selection Circuitry

**Figure 16.**
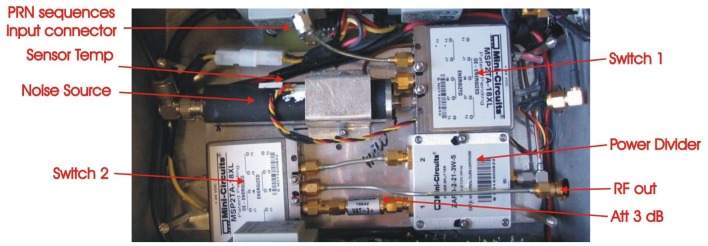
Hardware implementation of the selection circuitry from [6].

Once the two different noise sources (thermal and PRN) have been independently generated, these are injected into a selector circuitry (Figure 13). The function of this part is to select between the Noise Source or the PRN generator, and to provide two power levels so as to perform differential measurements. Figure 16 shows the hardware implementation. The first element (switch 1) is an absorptive mechanic switch model MSP2TA-18XL from MiniCircuits [30] to select between the two sources. It has an isolation of 100 dB at the frequency of operation. The selected source is divided into two branches through a power combiner model ZAPD-2-21-3W from MiniCircuits [31]. One of the branches is attenuated 0 dB and the other one is attenuated 3 dB. Finally a second absorptive mechanic switch (switch 2) selects the required attenuated output.

###### 3.2.6.2.2. Correlated Noise Unit Integration

**Figure 17.**
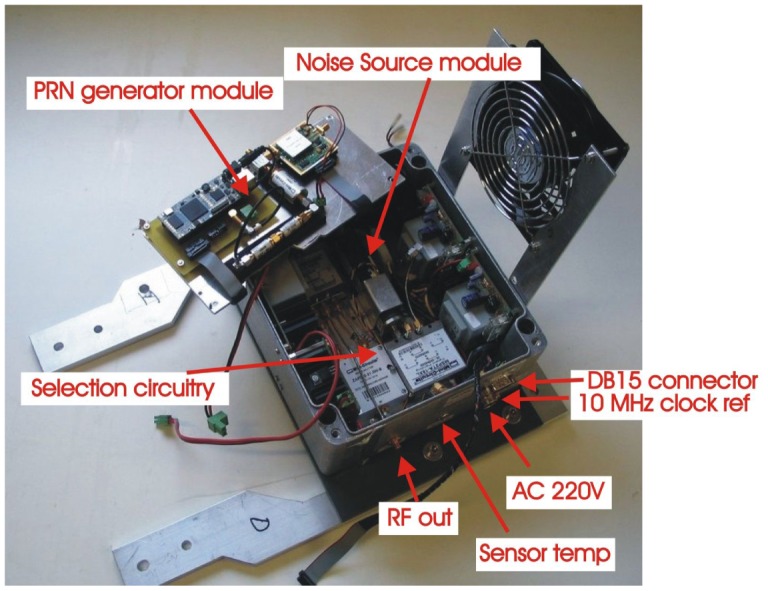
Hardware implementation of the correlated noise unit from [6].

Figure 17 shows the metallic box where the correlated noise sources and the selection circuitry have been integrated.

In order to minimize the area these modules have been integrated in different layers. In the bottom layer the Noise Source module and the selection circuitry have been implemented and the PRN sequences module in the top layer. The FPGA 2 synthesizes the PRN sequences. It is controlled with a DB15 connector through the internal PC as shown in Figure 17. Three commands are required to fully control the correlated noise unit (Figure 13): one to select the attenuation (0 or 3 dB), another one to select the correlated noise (Noise Source or PRN), and the last one to select the SR of the PRN.

#### 3.2.7. General Description of the Mobile Unit

Before starting with the design of the mobile unit it has been necessary to define a set of specifications in order to establish the necessary requirements to transport the PAU-SA and the Multi-frequency Experimental Radiometer With Interference Tracking For Experiments Over Land And Littoral (MERITXELL) [32] instruments. In order to transport the instruments, it has been necessary to choose the mobile unit. Since the two instruments have a considerable mass and volume, a NISSAN ATLEON 8.19.3 truck with a maximum weight of eight tons was selected. The elevator tower for measurement purposes is eight meters height and it has azimuth and elevation movements 0° ≤ *0* ≤ 150° and −180° ≤ *φ* ≤ +135°. It is compatible to work with both instruments, but only one is able to be operated while the other one is parked. The elevator tower has four positions: up or measuring, down or parked, calibration or looking to the internal absorber wall, and change the radiometer. All these commands are sent through the external computer, and finally controlled via a Programmable Logic Controller (PLC) located in the control panel in the truck. Moreover, the mobile unit has four stabilization legs manually controlled covering the maximum surface allowing to work with an instrument at eight meters high, under wind speed conditions up to 100 km/h. Both the elevator tower and the stabilization legs work with a hydraulic unit. In addition to providing the truck with an elevator tower for measurement purposes, it has an enclosure to storage and transport the instruments. A microwave absorber wall has been placed inside the mobile unit for calibration purposes, one for each instrument.

## 4. PAU-SA's Processing Implementation

The Digital Correlation Unit (DCU) computes all possible cross-correlations between antenna pairs at each polarization by counting the number of samples with the same sign (1 bit), obtaining the so-called correlation counts matrices (*N_cm,n_*). Figure 11 shows a single polarization correlation counts matrix structure: the upper triangle part of the matrix contains the I/I correlations between pairs of signals (real part of the visibilities samples), while the lower triangular part contains the I/Q correlations (imaginary part of the visibilities samples). The diagonal contains the cross-correlations of the I and Q components of the signals from the same antenna. These cross-correlated are related in MIRAS to the quadrature errors, but in PAU-SA, since the I/Q demodulation is performed digitally, there are no quadrature errors, and the diagonal is always zero. This 25 × 25 matrix is then completed by adding a right column containing the cross-correlation between the in-phase component and 0's (I-‘0’), and the bottom row with the cross-correlation between the quadrature component and 0's (Q-‘0’) (both for calibration purposes). These values are used to compensate for threshold errors in the comparators. The total number of samples *N_cmax_* is added at the right bottom element, which is used to normalize the whole *N_cm,n_* matrix. It can be observed that the number of correlation counts is an integer, with respect the equation: 0 < *N_cm,n_* < *N_cmax_*. Recalling that the sampling frequency (*F_S_*) is 5.745 MHz, and that the maximum integration time (*T_int_*) is 1 s, then the maximum number of counts or samples (*N_cmax_*) is:

(26) $$\begin{array}{cc} N_{c_{\text{max}}}=F_{S}\cdot T_{\text{int}}=5.745 & \text{M Samples} \end{array}$$

Finally, these matrices, and the power measurements are sent to an external PC in real time, where the calibration and the image reconstruction algorithms are implemented to retrieve the brightness temperature image.

The first step consists of computing the cross-correlation (c̿) by normalizing the correlation count matrix *N_cm,n_* to the maximum number of possible samples (*N_cmax_*), at each polarization (V, H and V/H):

(27) $$c_{m,n}=\frac{N_{c_{m,n}}}{N_{c_{\text{max}}}}$$

where *m* and *n* indicate the corresponding receivers. Once *c̿* is computed, it is necessary to compensate the offset introduced in the measurements by the up-counters and apply a scaling factor to obtain the digital correlation (Z and *μ* correspond to either the real or imaginary parts of the digital and the normalized cross-correlation, depending if the cross-correlation is performed between the in-phase components, or between the in-phase and quadrature components.) (*Z*):

(28) $$Z_{m,n}=A(c_{m,n}-{\text{offset}}_{m,n})$$

The digital correlation (*Z*) is a real number, for which it can be assumed that 0 < *Z* < 1. On one hand, when two signals are uncorrelated, the correlation *c_m,n_* = 0.5 and *Z_m,n_* = 0, and on the other hand, when two signals are correlated, the correlation *c_m,n_* = 1 and *Z_m,n_* = 1, therefore in the absence of hardware errors [33]

(29) $$Z_{m,n}=2\left(c_{m,n}-\frac{1}{2}\right)$$

The normalized cross-correlation (*μ*) between two Gaussian signals sampled with 1 bit (2 levels) is related to the digital correlation by the following expression:

(30) $$\mu_{m,n}=\sin\left(\frac{\pi }{2}Z_{m,n}\right)$$

However, this relationship is only valid for ADCs having a zero offset (ideal case). Taking into account the sample threshold error, to correct the correlation offset, the normalized correlation *μ* is calculated with a non-linear relationship (Equation (31)) using Equation (30) as initial solution, and using an iterative method (fixed point iteration or Newton-Raphson) [34,35] until the difference between two iterations is smaller than, for example, 10^−6^.

(31) $$\mu_{m,n}=\sin\left(\frac{\pi }{2}\left(Z_{m,n}+\frac{2}{\sqrt{1-\mu_{m,n}^{2}}}(\mu_{m,n}X_{01}^{2}+\mu_{m,n}Y_{01}^{2}-2X_{01}Y_{01})\right)\right)$$

In Equation (31)*X*_01_ is calculated in the last column of the matrix of correlators (26^th^ column) as the cross-correlator between the in-phase components (*I*) and a signal equal to all zeros. Similarly, *Y*_01_ is computed in the last row (26^th^ row) as the cross-correlator between the quadrature components (*Q*) and the signal to all zeros. After the fixed-point iteration, the new normalized correlation matrices are reorganized to compute the 25 × 25 visibility matrices. The upper and bottom parts of the diagonal are combined to form the normalized complex cross-correlation.

(32) $$\mu_{m,n}=\mu_{m,n}^{ii}-j\cdot \mu_{m,n}^{iq}=\mu_{m,n}^{ii}+j\cdot \mu_{m,n}^{qi}$$

where the subscripts stand for the correlation between two in-phase *ii*, and quadrature in-phase *iq* contributions. This relationship can be also be expressed as:

(33) $$\mu_{m,n}=\frac{1}{\sqrt{T_{SYS_{m}}T_{SYS_{n}}}}\left(ℜe\left[{\tilde{r}}_{m,n}^{ii}(0){\hat{V}}_{m,n}\right]+j\cdot ℑm\left[{\tilde{r}}_{m,n}^{qi}(0){\hat{V}}_{m,n}\right]\right)$$

where ℜe and *j*m are the real and imaginary part operators respectively, and ${\tilde{r}}_{m,n}^{ii}(0)$ and ${\tilde{r}}_{m,n}^{qi}$ are the Fringe-Wash Function (FWF) at the origin for the corresponding pair of receivers indicated by the sub-scripts, and the system temperatures of the denominator are given by:

(34) $$T_{\text{SYS}}≜T_{A}^{\prime }+T_{\text{REC}}$$

where $T_{A}^{\prime }$ is the antenna temperature including the contribution from the ohmic losses $\eta_{\Omega }(T_{A}^{\prime }=\eta_{\Omega }T_{A}+(1-\eta_{\Omega })T_{ph})$, and *V̂_m,n_* represents the corrected visibility: the *m* and the *n* receiver assuming that both receivers are at the same physical temperature *T_ph_*, which is given by:

(35) $${\hat{V}}_{m,n}=\int \int_{\xi^{2}\pm \eta^{2}\leq 1}\frac{T_{B}^{qp}(\xi ,\eta )}{\sqrt{1-\xi^{2}-\eta^{2}}}\cdot \frac{F_{m}(\xi ,\eta )}{\sqrt{\Omega_{m}}}\frac{F_{n}(\xi ,\eta )}{\sqrt{\Omega_{m}}}\cdot {\bar{\tilde{r}}}_{m,n}\left(-\frac{u_{m,n}\xi +v_{m,n}\eta }{f_{0}}\right)e^{-j2\pi (u_{m,n}\xi +v_{m,n}\eta )}d\xi d\eta$$

where the overbar in the FWF means normalization at the origin, that is:

(36) $${\bar{\tilde{r}}}_{m,n}(t)=\frac{{\tilde{r}}_{m,n}^{\alpha \beta }(t)}{{\tilde{r}}_{m,n}^{\alpha \beta }(0)}$$

Once the normalized complex cross-correlations *μ_m,n_* have been calculated for every combination of antenna polarizations, they are arranged as 25 × 25 visibilities matrices. Then, the normalized visibility function can be derived from Equation (37).

(37) $$V_{m,n}=\mu_{m,n}\cdot \sqrt{T_{SYS_{m}}T_{SYS_{n}}}$$

Finally, the visibility samples must be corrected for phase and amplitude errors [10,36]. The main differences with the MIRAS instrument calibration are:

1. Since the I/Q demodulation is performed digitally, quadrature errors are zero and do not have to corrected.
2. System temperatures are measured with digital PMS, therefore they are insensitive to offset and slope drifts as opposed to their analog counter parts,
3. Visibility offsets are measured with an internal matched load and by looking to an external absorber, and
4. Phase and amplitudes of the visibility samples are measured using either a centralized noise source or a pseudo-random noise sequence PRN [36].

(38) $${\hat{V}}_{m,n}=\mu_{m,n}\cdot \underset{\text{Amplitude calibration}}{\underset{︸}{\underset{\text{De}-\text{normalization}}{\underset{︸}{\sqrt{T_{SYS_{m}}T_{SYS_{n}}}}}\frac{1}{g_{m,n}}}}\underset{\text{Phase calibration}}{\underset{︸}{e^{j\alpha_{m,n}}}}$$

Afterwards, these visibility samples must be re-ordered and assigned to the corresponding baseline ((*u, v*) point) before applying the image reconstruction algorithm. Since it has not been possible to measure the antenna patterns when mounted in the structure, antenna pattern mismatches are compensated for by using a sort of Flat Target Response as described in [10].

## 5. Inter-Comparison between MIRAS and PAU-SA

Table 1 shows an inter-comparison table between both instruments to show up the potential improvements over the current MIRAS design that have been implemented and tested.

One of the main differences between MIRAS and PAU-SA is the frequency of operation. L-band radiometer should operate in the 1,400–1,427 MHz “protected” band as MIRAS. However, PAU-SA is an instrument concept demonstrator, and to minimize the hardware requirements the GPS reflectometer and the L-band radiometer share the same front-end and frequency band. Although sharing the same front-end provides a significant hardware reduction, there are some drawbacks. The first one concerns to the possible interference that GPS signals can introduce in the radiometric measurements. This non-optimal operation frequency for the radiometer instrument has an impact on the radiometric measurements, introducing some errors. This issue is analyzed in Section 5.1. The second one concerns the bandwidth. In the case of MIRAS, the bandwidth is limited by the protected band with a maximum value of 27 MHz, and an effective noise bandwidth of ∼19 MHz. For the PAU-SA instrument the bandwidth is 2.2 MHz imposed by the IF frequency of the GP2015 chip from Zarlink being used, and by the SAW filters used for the implementation of the receiver chain. This reduction in the bandwidth has an impact on the radiometric sensitivity that can only be compensated by increasing the integration time, which is not critical in a ground-based instrument.

Each arm of the MIRAS instrument is approximately three times longer than the PAU-SA ones: ∼4 m with 23 elements in front of 1.3 m with just 8 elements. The total number of antennas in MIRAS is 69 and 25 in the case of PAU-SA. This decision was taken for two reasons: the first one is due to the use of fixed non-foldable arms in order to simplify the mechanical complexity, and the second one was pragmatic: to be able to take the instrument out of the laboratory, where it was assembled. Since the bandwidth of PAU-SA is narrower than that used in MIRAS and the arm length is smaller, spatial decorrelation effects modeled by the FWF are negligible. This factor is quantified in Section 5.2. One of the improvements of PAU-SA is the additional dummy antenna at the end of each arm to improve the antenna pattern similarity (Figure 2).

Concerning the antenna type and separation, they are quite similar in both instruments. For instance, both MIRAS and PAU-SA use patch antennas without dielectric substrate for the V- and H-polarizations. For hardware simplicity, MIRAS has sequential acquisitions since the receiver is shared for both polarizations. In the case of PAU-SA, each polarization has its own receiver channel. Therefore, continuous acquisitions at both polarizations can be obtained simultaneously, as well as mixing the polarizations.

In order to increase the AF-FOV, the minimum distance between element spacing is kept to the minimum, only limited by the receiver's size. MIRAS has an antenna spacing of 18.75 cm, corresponding to 0.875 λ at 1,400 MHz. In the case of PAU-SA the antenna spacing is reduced to 15.5 cm = 0.816 λ at 1,575.42 MHz, the GPS L1 signal.

Moreover, the distribution of the LO used in the down-converter is different in both instruments. In MIRAS the LO is fed to groups of 6 elements forming an arm section, whereas PAU-SA uses a centralized reference clock of 10 MHz, and the LO is generated by means of a PLL inside each receiver to minimize offsets coming from common LO noise leaking through the mixer.

MIRAS'quantification scheme uses 1 bit sampling at intermediate frequency IF [37] depending upon the noise uptake level inside the Light Cost Effective Front-end (LICEF). PAU-SA uses 8 bits ADC using IF sub-sampling techniques to down-convert and demodulate simultaneously. Using 8 bits, it is possible to filter, demodulate, and perform the power estimation, and finally use only 1 bit to obtain the three complex correlation matrices V, H, V/H.

Due to the large number of receivers in interferometric radiometers, it is advisable to obtain a quasiperfect matching of the frequency responses, mass reduction, and to eliminate temperature and frequency drifts as much as possible. For these reasons, the most important contributions in PAU-SA are focused on the replacement of analog by digital subsystems being the most important:

- I/Q down-conversion to eliminate quadrature errors,
- Digital filtering, replacing the narrow RF filter by a digital IF filter, to obtain a mass reduction, a quasi perfect matching, and eliminating thermal and frequency drifts, and
- Digital Power estimation, eliminating the classical Schottky or tunnel diodes to achieve a mass reduction, and eliminating temperature drifts and aging.

All these subsystems and the DCU that computes the full cross-correlation matrix (V, H and V/H) have been implemented in a Virtex 4 FPGA. In this case, since the clock frequency is much higher than the sampling frequency, hardware reuse techniques are used to compute the full-polarization matrices in each snapshot.

The imaging capabilities can be sequential dual-polarization or full-polarimetric in MIRAS, and non-sequential full-polarimetric in PAU-SA. The use of both polarizations simultaneously is also necessary to compose the reflected LHCP GPS signal.

The integration time is fixed for MIRAS with a value of 1.2 s, while PAU-SA has predefined 4 values: 10 ms, 100 ms, 0.5 s and 1 s for test purposes.

Finally, MIRAS uses a classical correlated noise injection method for calibration proposes. Due to the large number of receivers to feed, it is necessary to use several noise sources distributed along the instrument increasing the hardware complexity, and introducing additional noise. In addition, the distributed noise injection is not capable of calibrating all sorts of errors. To overcome this problem PRN signals can be used instead of a centralized noise source for calibration purposes. Moreover, since the PRN signals are deterministic and known, new calibration approaches are feasible through the correlation of the output signals with a local replica of the PRN signal, leading to the estimation of the receivers' frequency responses and the FWF [36]. PAU-SA has the possibility of using both the classical noise-injection method or this new technique with PRNs.

### 5.1. Impact of the Frequency Operation on the Radiometer Part

**Figure 18.**
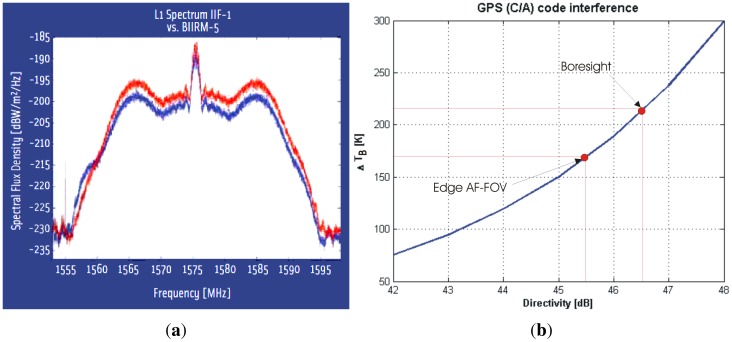
(**a**) L1 ACF of the P code (in blue), and the C/A code (in red) from [38], (**b**) estimated Δ*T_B_* contribution in the systematic *T_B_* image due to GPS C/A code in the PAU-SA's radiometric part as a function of the GPS position in the FOV from [6].

The receiver's operating frequency is defined by the L1 signal of the GPS signal (1,575.42 MHz), which is also suitable for SSS estimation. On one hand the GNSS signals use spread spectrum techniques. After the scattering on the sea surface, the power of these signal is at least 23 dB below the thermal noise. For this reason, and thanks to the 30.1 dB correlation gain, GNSS-Reflectrometry can detect the GPS signal when the correct C/A code is applied. On the other hand, from the radiometer point of view, the noise signal to be detected is at least 23 dB above the GPS signal so the radiometric error induced is minimum, and it only occurs in the directions of specular reflection which are known a prior. Therefore, it is possible that both the radiometer and the reflectometer share the same receiver. Although the spread-spectrum of the GPS modulation is at least 23 dB below the noise level (PAU-SA as a radiometer), its impact has to be quantified specially since the synthesized beam is very narrow. The GPS signal includes the P, C/A and M codes. Figure 18(a) shows the auto-correlation function (ACF) of P and C/A codes.

The first one is the P code, having the largest repetition period of 6.1871 × 10^12^ bits long (6,187,100,000,000 bits, ∼773.39 gigabytes) repeating once a week, and achieving a ∼75 dB compression gain with a 22 MHz band, and the received power level is ∼−133 dBm (−143 dBm in the ∼2.2 MHz C/A code bandwidth) [39]. The C/A code has a period of 1,023 bits, repeating every 1 ms, and achieving a 30 dB of compression gain with a 2.2 MHz, and the received power level is ∼−130 dBm. M code is still experimental and, in addition, it distributes its power at the edges of the band (Figure 18(a)), having less effect even than the P code. From the point of view of the radiometric measurements, the correlation gain does not have any effect, and only matters the power density associated at each code. For this reason the GPS signal to be taken into account is the C/A code, being 10 dB higher than the P code. To measure the impact of the GPS signal in the PAU-SA's radiometric measurements, the PAU-SA's antenna pattern presented in the previous chapter has been analyzed in two extreme cases: the worst case is when the specular reflection point is at the antenna's boresight (maximum directivity of the synthetic antenna pattern: 46.5 dB), and the best situation comes from the interference at the edge of the AF-FOV (minimum directivity of the synthetic antenna pattern: 45.5 dB), only 1 dB below the maximum. Figure 18(b) shows the contribution of the GPS signal (C/A code) to the radiometric measurements Δ*T* considering a sea surface reflection coefficient of Γ = 0.7, and the PAU-SA system parameters. As it can be appreciated, independently of where the interference comes from, the GPS signal has a high contribution to the radiometric measurements (between 170 K and 220 K respectively). This means that, in the case of PAU-SA, to perform radiometric measurements the instrument must be pointed to the North, where the density of GPS satellites is minimum due to their orbital plane distribution. Moreover, if a GPS satellite interferes in the radiometric measurements, it will appear as a “point source” in the retrieved image, which could be subtracted by making measurements at different time. (GPS satellites will appear at a different position).

### 5.2. Impact of the Spatial Decorrelation Effects in the Visibility Function

The Fringe-Wash Function FWF accounts for the spatial decorrelation of the signals coming from a given direction at a given baseline. Precisely, the FWF is related to the frequency responses of the receivers forming the baseline.

The amplitude of the FWF can be modeled around the origin by means of a sine function as:

(39) $$\left|G_{kj}(\tau )\right|\approx A\cdot \text{sinc}(B\cdot (\tau -C))$$

where *A* · *sinc*(*B* · *C*) is the amplitude at *τ* = 0, *B* is the noise bandwidth, and *C* is the value of *τ* in which the fringe-washing function is maximum. The transit time *τ* from given (ξ, η) directions to the antennas forming the baseline (*u, v*) is

(40) $$\tau =-\frac{u\xi +v\eta }{f_{0}}$$

**Figure 19.**
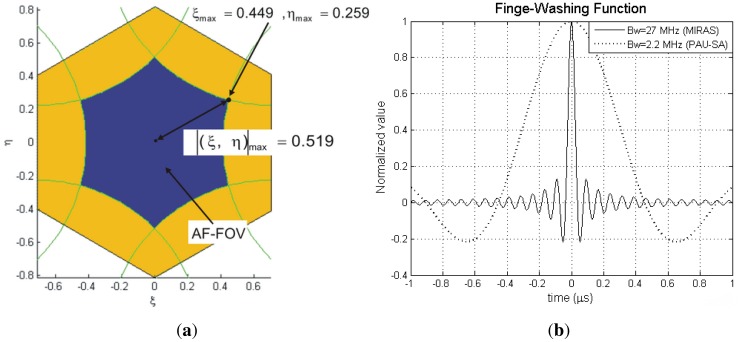
(**a**) PAU-SA's AF-FOV showing the maximum distance in the plane (ξ, η), and (**b**) FWF comparison between MIRAS and PAU-SA instruments. (from [6]).

The maximum value is found for the largest baseline Δ*u_max_*, and the (ξ, η) direction distance farthest away from the boresight within the AF-FOV, |(*ξ, η*)|*_max_* = 0.519 (Figure 19(a)).

The maximum antenna separation is given by:

(41) $$\Delta u_{\text{max}}=2\sqrt{3}N_{EL}d$$

where *N_EL_* is the number of antennas in each arm and *d* is the distance between adjacent antennas. In the case of PAU-SA *N_EL_* = 8, *d* = 0.816 *λ*, and Δ*u_max_* = 22.61 *λ*. Figure 19(b) shows a comparison between the FWF of MIRAS and PAU-SA systems. In this case, since the bandwidth in MIRAS is about 10 times larger than PAU-SA, and the array size is approximately 3 times larger than PAU-SA, the FWF is ∼30 times narrower in MIRAS than in PAU-SA. As it can be appreciated in Figure 19(b), the PAU-SA's FWF in the evaluated range is very close to one (FWF ∼ 0.9995), so that this parameter is negligible in the PAU-SA system.

## 6. Instrument Characterization and Experimental Results

This section is divided into three parts. The first part presents the temperature characterization of the instrument, the second part is the characterization at baseline level in an anechoic chamber, and the last part is an outdoor experiment with the instrument.

### 6.1. Thermal Control Characterization

**Figure 20.**
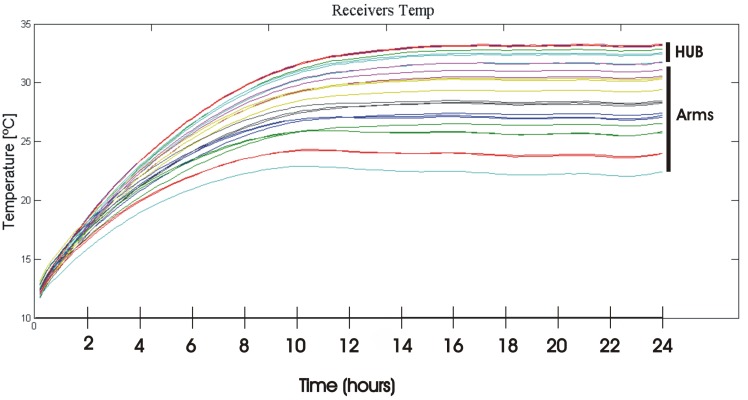
Sample time evolution of the PAU-SA's receivers acquired during the day (2011-02-15) from [6].

Since receivers exhibit phase and amplitude drifts due to temperature changes, it is necessary to stabilize and control the temperature of the instrument. The better the temperature control, the longer the inter-calibration period will be. The PAU-SA's stability temperature is controlled by Proportional Integral Derivative (PID) devices located in each arm, and in the central part or hub. Moreover, it has an air control by means of fans to distribute the air along the instrument and forcing the air circulation as much as possible, but in the case of the HUB it is not sufficient, being the warmest the central elements. Figure 20 shows a plot of the control temperature during one day long in a measurement campaign. As it can be appreciated, in the stable transition there is a temperature excursion of about 10 °C, however, the maximum standard deviation of each sensor which is acceptable is only 0.27 °C.

### 6.2. Measurements at Baseline Level

The tests performed at baseline level are: the characterization of the radiometer noise and its stability through the “Allan's variance” [40], and the characterization of the radiometer resolution.

#### 6.2.1. Radiometer Stability

**Figure 21.**
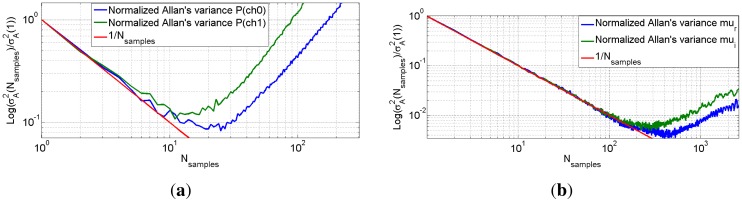
(**a**) Normalized power variance *versus* number of samples, (**b**) normalized correlation (real and imaginary parts) *vs.* number of samples. Pictures from [6].

In order to determine the optimum range of integration times for best use of the system, the characterization of the radiometer noise and stability has been performed measuring the Allan's variance [40] given by:

(42) $$\sigma_{A}^{2}(\tau )=\frac{1}{2}\langle {\left({\bar{u}}_{n+1}-{\bar{u}}_{n}\right)}^{2}\rangle$$

where *τ* is the integration time, and *μ̄_n_* is the *n^th^* fractional frequency average over the observation period. This method consists of the determination of the Allan's variance *versus* integration time allowing to determine the different types of fluctuations of the radiometer output signal. In particular, the range of integration times for the optimum setup of the calibration and measurement is shown in Figure 21.

It shows the evolution of the variance of both channels *versus* the number of samples. The ideal case (thermal Gaussian noise) must decrease monotonically decreasing function as:

(43) $$\sigma_{A}^{2}=\frac{1}{N_{\text{samples}}}$$

However, due to system instabilities, after a given value of *N*, the variance $\sigma_{A}^{2}$ increases again. Taking into account that a sample represents a measurement of 1 s, the optimum number of samples is the value of minimum variance and determines the maximum integration time that may be used, without degradation due to radiometer drifts. On one hand, Figure 21(a) plots the power variance *vs.* the number of samples. The maximum integration time for calibration purposes using correlated noise, and measuring the visibilities de-normalization procedures is 15 s. One the other hand, Figure 21(b) shows the normalized correlation *vs.* the number of samples to determine for phase and the ADC offset calibration. This time is ∼200 s. Therefore, gain fluctuations are dominant and are the ones that limit the maximum integration time.

### 6.3. Radiometer Resolution Validation

**Figure 22.**
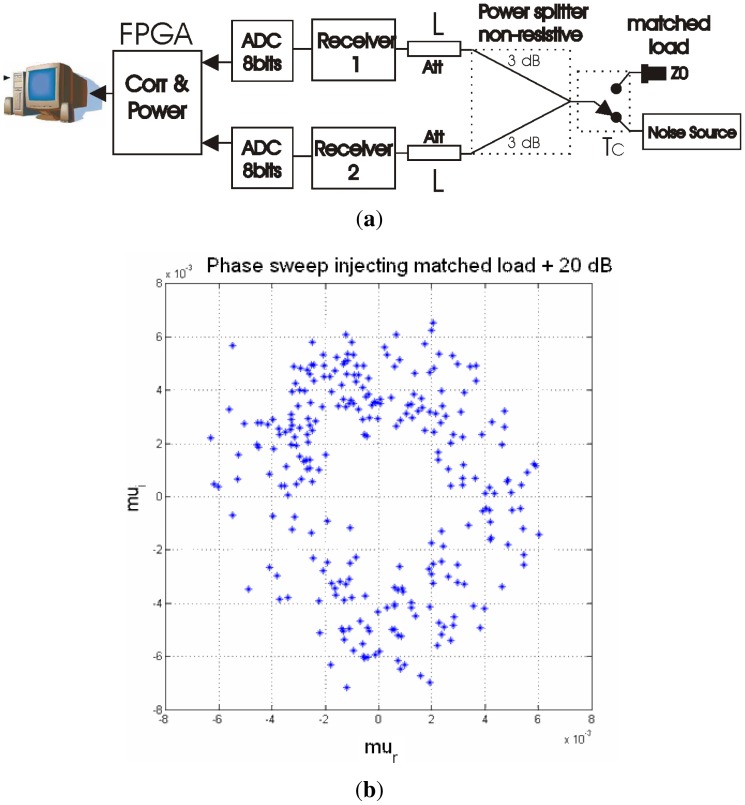
(**a**) Sensitivity measurement test set-up, and (**b**) measurement sensitivity circle with a matched load and an attenuation of 20 dB with an integration time of 1 s. Pictures from [6].

The characterization of the radiometric resolution has been performed with the set up shown in Figure 22(a). A noise source or a matched load are connected to the input of a non-resistive power splitter producing correlated noise. Two adjustable attenuators are connected to the power splitter outputs producing uncorrelated noise and attenuating the correlated noise generated by the matched load. The complex correlation is then measured for different values of the attenuation and for different phases. Phases have been randomly obtained by turning OFF and ON rapidly the PLL, so each time the receiver's PLL locks to a different phase.

Concerning the shape of the circle is clearly distinguished up to 2 × 20 dB attenuators, and since the I/Q demodulation is performed digitally, there are neither quadrature errors, nor amplitude unbalances between branches that need to be corrected [41]. The radius of the circles can be determined estimating the correlation *μ* of two digital signals (Equation (44)):

(44) $$\mu =\frac{\frac{T_{c}}{2L}}{T_{\text{SYS}}}=\frac{\frac{T_{c}}{2L}}{T_{A}^{\prime }+T_{\text{REC}}}$$

where *μ* is equal to the corresponding analog signal power (*T_c_*) normalized to the system temperature *T_SYS_*. Neglecting the outgoing noise waves from each receiver front-end that couple into the other receiver, *T_SYS_* can be estimated as (Equation (45)):

(45) $$T_{\text{SYS}}≅\frac{T_{c}}{2L}+T_{ph}\left(1-\frac{1}{L}\right)+T_{\text{REC}}$$

where *L* is the attenuation introduced, *T_ph_* is the physical temperature, and *T_c_* = 290 K (matched load). Evaluating the previous equations with *L* = 20 dB, *T_ph_* = 290 K, and *T_REC_* ∼ 250 K than, *T_SYS_* = 538.55 K, and the correspondent *μ* = 2.7 · 10^−3^, which is very close to the measured value (Figure 22(b)).

**Figure 23.**
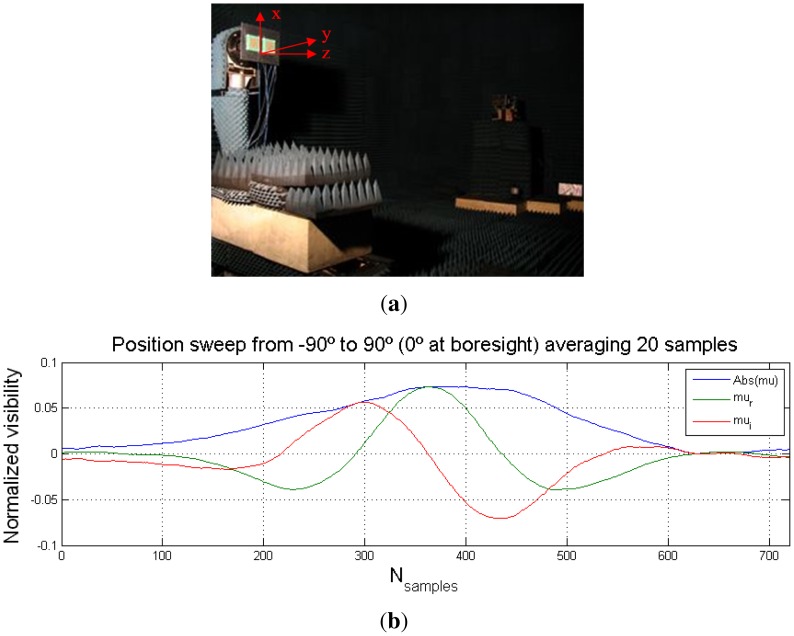

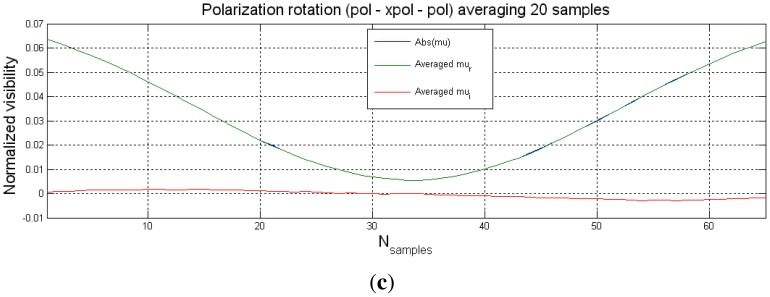
(**a**) Picture of the baseline level measurements during anechoic chamber test, and normalized correlation (real and imaginary) measurements with baseline rotating at (**b**) X-axis, and (**c**) Z-axis. Pictures from [6].

Moreover other tests have been performed in the anechoic chamber [42] to measure the normalized visibility by scanning the pairs of receivers from −90° to 90°, at the polarization from V to H as shown in Figure 23(a). Results are contrasted with theoretical results through Equation (4). Considering that the fringe-wash function is negligible (1/B ≪ maximum transit time) [43] and that both antenna patterns can be considered to be equal, the normalized visibility can be determined by:

(46) $$V(\theta )=\frac{P_{T}G_{T}(\theta )}{4\pi r^{2}}\frac{\lambda^{2}}{4\pi }D_{R}t(\theta )e^{-j2\pi \frac{d}{\lambda }\sin\theta }$$

In this situation, the theoretical real and imaginary normalized correlations should vary according to: real part in Equation (47) and the imaginary part in Equation (48) as shown in Figure 23(b).

(47) $$\mu_{r}=-\sin\left(\frac{2\pi d}{\lambda }\sin\theta_{0}\right)$$

(48) $$\mu_{i}=\cos\left(\frac{2\pi d}{\lambda }\sin\theta_{0}\right)$$

Due to the previous considerations, in Equation (46) the absolute value of the normalized visibility is the antenna radiation voltage pattern *t*(*θ*) when the transmitting antenna has an orthogonal polarization. Figure 23(c) shows that the normalized correlation sweeps the emitter with different polarization, in the center with opposite polarization.

### 6.4. Experimental Results

This section summarizes a couple of experimental results carried out with the PAU-SA instrument, calibration procedures and extended results are presented in detail in [10]. The instrument performance has been evaluated in terms of AF-FOV, angular resolution, radiometric resolution, radiometric precision, and radiometric bias. Moreover, since PAU-SA operates at the GPS L1 band, maps of the GPS satellites constellation have been obtained. To check at first glance the instrument's health, firstly a PRN source has been transmitted in order to confirm that the source was imaged in the right direction and also determine the AF-FOV.

**Figure 24.**
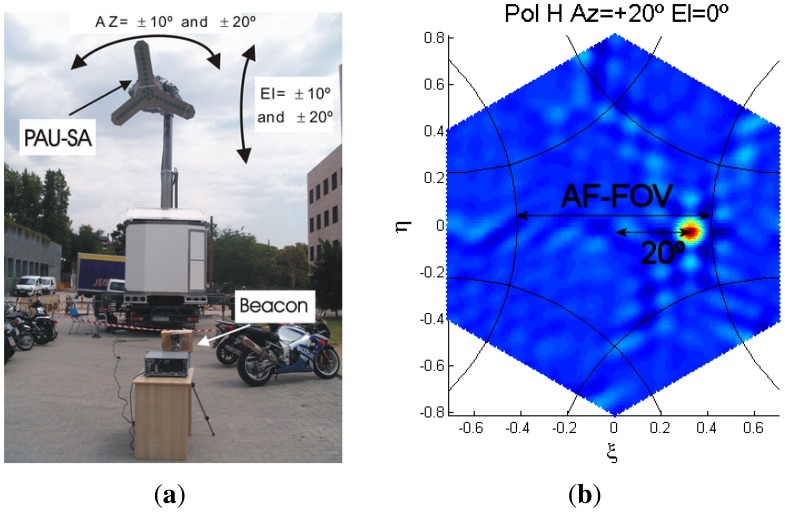
Measurement setup using a PRN signal as point sources at 10 m of the instrument. (**a**) single transmission moving the instrument in azimuth and elevations angles to determine the AF-FOV, and (**b**) recovered image moving the instrument 20° in azimuth an applying near-field to far-field compensation (from [6]).

Figure 24(a) shows the PAU-SA instrument in the top of the 8 m height robotic arm and the beacon on the ground. Since it is easier to move the instrument than the beacon, the PAU-SA instrument was moved ±10° and ±20° both in azimuth and elevation. Figure 24(b) shows one of the images retrieved when the instrument was moved 20° in azimuth, which can be compared with the theoretical one (Equation (49)), and determines the AF-FOV to be approximately around 48°.

(49) $$\frac{1}{2}\cdot \text{AF}-\text{FOV}=\arcsin\left(\frac{2}{\sqrt{3}d}-1\right)=24{}^{\circ}$$

The second test has been the determination of the angular resolution transmitting two point sources to the instrument and using a hexagonal inverse Fourier transform, and a rectangular window with an angular resolution of Δ*ξ* = 0.1 corresponding with 5.7°. The radiometric specifications have been achieved considering the same antenna pattern and neglecting the FWF and the Corbella's term:

- The radiometric resolution is the standard deviation of the time fluctuation of a given observable. It is the minimum change detectable by the instrument and it is computed as in Equation (50). It has been found to be *σ_v,h_* =1.9 K at both polarizations.
- The radiometric precision is the systematic error in each pixel. An average radiometric precision value is computed for the whole alias-free field-of-view image as the RMS value of the brightness temperature computed from the average of 80 snapshots of 3 s integration time each (total 240 s), so as to achieve a negligible radiometric resolution). It is estimated by using Equation (51) *σ_v_* = 1.2 K and *σ_h_* = 2.0 K, and
- The radiometric bias is the spatial mean of the computed brightness temperature minus a reference temperature determined by Equation (52). It is found to be −1.6 K and −1.8 K, at vertical and horizontal polarizations, respectively.

(50) $$\Delta T_{\text{resolution}}\approx \sqrt{\frac{\sum_{i=1}^{M}{\left({\hat{T}}_{B}(\xi ,\eta ,t_{i})-{\langle {\hat{T}}_{B}(\xi ,\eta ,t)\rangle }_{t}\right)}^{2}}{M-1}}$$

(51) $$\Delta T_{\text{precision}}\approx \sqrt{\frac{\sum_{i=1}^{N}{\left({\langle {\hat{T}}_{B}(\xi_{i},\eta_{i},t)\rangle }_{t}-{\hat{\bar{T}}}_{B}(\xi_{i},\eta_{i})\right)}^{2}}{N-1}}$$

(52) $$\Delta T_{\text{bias}}=\frac{1}{N}\sum_{i=1}^{M}\left({\langle {\hat{T}}_{B}(\xi ,\eta ,t_{i})\rangle }_{t}-T_{B}(\xi ,\eta )\right)$$

**Figure 25.**
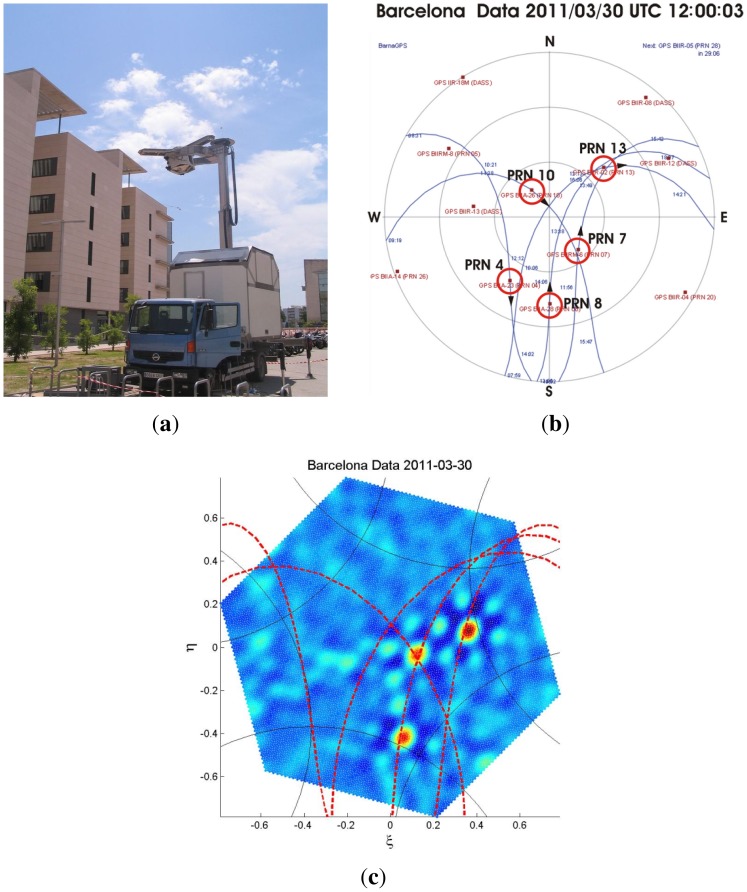
(**a**) PAU-SA pointing to the GPS satellites, (**b**) Map of GPS satellites' paths as seen from the test location on March 30th, 2011. Sequential of images recovered by PAU-SA every 44 min: (**c**) animation (click on the image) of the GPS satellites' movements UTC 12:00:03. Note: *ξ* = 0, *η* ≥ 0 corresponds to the geographic north from [6]).

Finally, since the instrument was conceived as a technology demonstrator, commercial GPS chips operating at L1 band were used, making it possible to track the GPS constellation. Figure 25(a) shows the instrument pointing to the sky seeking satellites, Figure 25(b) shows the map of the expected GPS satellites' paths in this time and location, and Figure 25(c) shows a video animation of the satellites' movements appearing as points sources and following the expected tracks.

## 7. Conclusions

This paper has described the PAU-SA instrument. It is a synthetic aperture radiometer that has been designed and tested to study potential improvements in SMOS follow-on or in other missions using synthetic aperture radiometers as GAS [44], PATH [45] or GIMS [46]. A comparison table between MIRAS and PAU-SA has been presented, describing the main novelties of the PAU-SA instrument design. These contributions have been focused on the replacement of analog by digital subsystems such as: I/Q down-conversion, digital filtering, full-correlation matrix (V, H and VH), power estimation implemented in an FPGA, and the use of both a centralized noise source, and PRN signals for calibration purposes. This last one has been one of the most remarkable contributions in the hardware design, since with this method it is possible to feed a large number of receivers using a centralized topology. Once the instrument has been assembled and calibrated, imaging results can be obtained. Instrument characterization has focused on the measurement of Allan's variance, the angular resolution, and the radiometric performance. Finally, imaging results recovery of point sources using PRN sequences and imaging real GPS satellites have been performed. However, the radiometric performances achieved so far are not good enough for the sea surface salinity retrievals originally foreseen.

## Figures and Tables

**Table 1.** Comparative between MIRAS and PAU-SA. Table from [6].

**N**°	**Parameter**	**MIRAS/SMOS**	**PAU-SA**	**Comments**
1	Altitude	Global observation, Low Earth Orbit (LEO): orbital altitude of 763 km, 3 days equatorial revisit time	On-ground	

2	Frequency operation	L-band (1,400–1,427 MHz) band is protected for passive observations	L1-band (1,575.42 MHz) GPS signal	Same frequency for Radiometer and GNSS-Reflectrometer

3	Bandwidth	19 MHz	2.2 MHz	Negligible spatial correlation effects

4	Number of antennas per arm	4 m	1.3 m	

5	Number total antennas	69	31	8 × 3 + 1 = 25 for Radiometer, 3 center plus 3 additional = 7 antennas for GNSS-Reflectometer, 3dummy antennas, 1 at the end of each arm

7	Antenna type	Patch antenna without dielectric substrate and V & H polarizations (non-simultaneous)	Patch antenna without dielectric substrate and V & H polarizations (simultaneous)	Full-polarimetric (non-sequential)

8	Antenna spacing	0.875λ at 1,400 MHz, 21 cm wavelength	0.816λ at 1,575.42 MHz, 19 cm wavelength	Increase the alias-free field of view

9	Receiver type	1 per element	1 per polarization (2 per element)	Full-polarimetric possible (non-sequential)

10	Topology of the LO down-converter	Distributed local oscillator (LO) (groups of 6 elements)	Centralized reference clock + Internal LO generator	Elimination of correlation offsets due to LO noise leakage.

11	Quantization	1 bit IF sampling depending upon the noise uptake level (Inside the LICEF)	8 bit IF sub-sampling using an external ADC	(8 bits) for I/Q conversion and (1 bit) to power measurement

12	I/Q down-conversion	Analog	Digital	Mass reduction, no quadrature errors (calibration not required)

13	Frequency response shaped by	Analog RF filter	Digital low- pass filter	Mass reduction, quasi perfect matching, no temperature and frequency drifts

14	Power measurement system (PMS)	Analog (diode detector)	Multibit Digital (FPGA) Computation	Mass reduction, no temperature drifts

15	Digital Correlated Unit	*F_CLK_* =*F_s_*	*F_CLK_* ≫ *F_s_*	Clock frequency (*F_CLK_*) much higher than sampling frequency (*F_s_*) allows hardware reuse and compute full-polarimetric correlation matrices in one snapshot inside FPGA

16	Image capabilities	Dual-polarization or full-polarimetric (sequential)	Full-polarimetric (non-sequential)	Necessary for GNSS-R applications

17	Integration time	1.2 s	Variable: 4 values 1 s, 0.5 s, 100 ms, 10 ms	

18	Correlated Noise Injection	Distributed (Noise Source)	Centralized (Noise Source, or PRNs)	Using PRNs independent number of receivers (simpler and more flexible calibration)
